# Genome-wide identification of AP2/ERF transcription factor-encoding genes in California poppy (*Eschscholzia californica*) and their expression profiles in response to methyl jasmonate

**DOI:** 10.1038/s41598-020-75069-7

**Published:** 2020-10-22

**Authors:** Yasuyuki Yamada, Shohei Nishida, Nobukazu Shitan, Fumihiko Sato

**Affiliations:** 1 Laboratory of Medicinal Cell Biology, Kobe Pharmaceutical University, Kobe, Japan; 2 Division of Integrated Life Science, Department of Plant Gene and Totipotency, Graduate School of Biostudies, Kyoto University, Kyoto, Japan; 3 Graduate School of Science, Osaka Prefecture University, Sakai, Japan

**Keywords:** Molecular biology, Plant sciences

## Abstract

With respect to the biosynthesis of plant alkaloids, that of benzylisoquinoline alkaloids (BIAs) has been the most investigated at the molecular level. Previous investigations have shown that the biosynthesis of BIAs is comprehensively regulated by WRKY and bHLH transcription factors, while promoter analyses of biosynthesis enzyme-encoding genes have also implicated the involvement of members of the APETALA2/ethylene responsive factor (AP2/ERF) superfamily. To investigate the physiological roles of AP2/ERF transcription factors in BIA biosynthesis, 134 *AP2/ERF* genes were annotated using the draft genome sequence data of *Eschscholzia californica* (California poppy) together with transcriptomic data. Phylogenetic analysis revealed that these genes could be classified into 20 AP2, 5 RAV, 47 DREB, 60 ERF and 2 Soloist family members. Gene structure, conserved motif and orthologous analyses were also carried out. Gene expression profiling via RNA sequencing in response to methyl jasmonate (MeJA) indicated that approximately 20 *EcAP2/ERF* genes, including 10 group IX genes, were upregulated by MeJA, with an increase in the expression of the transcription factor-encoding gene *EcbHLH1* and the biosynthesis enzyme-encoding genes *Ec6OMT* and *EcCYP719A5*. Further quantitative RT-PCR confirmed the MeJA responsiveness of the *EcAP2/ERF* genes, i.e., the increased expression of 9 group IX, 2 group X and 2 group III ERF subfamily genes. Transactivation activity of group IX *Ec*AP2/ERFs was also confirmed by a luciferase reporter assay in conjunction with the promoters of the *Ec6OMT* and *EcCYP719A5* genes. The physiological roles of *AP2/ERF* genes in BIA biosynthesis and their evolution in the regulation of alkaloid biosynthesis are discussed.

## Introduction

Plants produce structurally divergent low-molecular-weight chemical compounds commonly classified as phenylpropanoids, aromatic polyketides, terpenoids and alkaloids to protect themselves against biotic and abiotic stresses such as pathogen or herbivore attack and UV irradiation. These compounds are also used as important pharmaceuticals, essential oils and flavourings; however, some of them are not easy to acquire because their distribution is limited to specific plant species, and their accumulation in plants is often very low.

Benzylisoquinoline alkaloids (BIAs) constitute a large group of alkaloids and include many pharmaceutically useful compounds, e.g., the analgesic morphine, the antitussive codeine, and the antimicrobial agents berberine and sanguinarine^1^. Although BIAs are found mainly in the Papaveraceae, Ranunculaceae, Berberidaceae, and Menispermaceae families^2^, the biosynthetic pathways of BIAs, especially berberine, sanguinarine and morphine in *Coptis japonica* (Ranunculaceae), *Eschscholzia californica* (Papaveraceae) and *Papaver somniferum* (Papaveraceae), respectively, have been intensively investigated at the molecular level^3,4^. Biosynthetic enzyme-encoding genes have been largely identified and characterized; furthermore, transcription factors involved in the regulation of BIA biosynthesis have also been isolated, e.g., WRKY superfamily transcription factors such as *Cj*WRKY1 from *C. japonica* and *Ps*WRKY from *P. somniferum* and basic helix-loop-helix (bHLH) transcription factors such as *Cj*bHLH1 from *C. japonica* and *Ec*bHLH1-1/1-2 from *E. californica*^5–8^. In particular, both *Cj*WRKY1 and *Cj*bHLH1 function as comprehensive transcription factors; that is, they upregulate the expression of nearly all BIA biosynthesis enzyme-encoding genes in *C. japonica* cells through direct interaction with their promoter region^6,9^.

Transcription factors are powerful tools that can be used for enhancing overall gene expression for the biosynthesis of valuable metabolites^10,11^. For example, the ectopic expression of two transcription factors involved in anthocyanin biosynthesis isolated from snapdragon, Del and Ros1, strongly improves the production of anthocyanins in tomato^12^. However, a marked increase in metabolite production by simple overexpression of transcription factors is not often observed because their activity is generally fine-tuned via direct or indirect interaction with multiple transcriptional regulators, and simple overexpression cannot overcome the limitations of biosynthetic flux. Furthermore, the application of transcription factors involved in BIA biosynthesis is more limited compared to that in phenylpropanoid biosynthesis due to a lack of information about the regulatory network of the former. In fact, the ectopic expression of *Cj*WRKY1 in cultured *E. californica* cells modulates the expression of BIA biosynthesis enzyme-encoding genes, resulting in increased accumulation of some BIAs, though the accumulation level increased by only 3- to 4-fold^13^.

To elucidate the detailed regulatory mechanism of gene expression and investigate other transcription factors in BIA biosynthesis, the characterization of *cis*-acting elements in the promoter region of BIA biosynthesis enzyme-encoding genes was performed in *C. japonica* cells^9^. This analysis revealed that a GCC-box-like sequence that may be a target of APETALA2/Ethylene responsive factor (AP2/ERF) family proteins in the promoter of BIA biosynthesis enzyme-encoding genes is possibly involved in the regulation of BIA biosynthesis. Further, ectopic expression of *Arabidopsis thaliana* and *Glycine max* AP2/ERF transcription factors in *E. californica* and *P. somniferum* cells increased the transcript levels of several BIA biosynthesis enzyme-encoding genes and influenced BIA production^14^.

The AP2/ERF superfamily is one of the largest groups of plant-specific transcription factors, and the members are defined by a conserved AP2/ERF domain that consists of 60 to 70 amino acid residues and is involved in DNA binding. The AP2/ERF superfamily can be divided into three subfamilies: AP2, RAV and ERF^15–17^. The AP2 family proteins mainly contain two AP2/ERF domains, and the others contain a single AP2/ERF domain. The RAV family proteins also contain a B3 domain. Furthermore, the ERF family can be divided into the DREB subfamily, whose members are well known to be involved in drought tolerance, and the ERF subfamily, whose members are involved in various stress responses^15,16^. Numerous reports have revealed the important role of AP2/ERF transcription factors; e.g., the AP2 and RAV family proteins are involved in plant growth and flower development, and the DREB and ERF subfamily members play an important role in biotic and/or abiotic stress responses, disease resistance, and plant hormone signalling and crosstalk such as ethylene, abscisic acid and jasmonate (JA)^18^. In the regulation of secondary metabolism, many AP2/ERF family transcription factors belonging to the ERF subfamily have also been confirmed to have a key role under JA-responsive signalling, especially nicotine and monoterpenoid indole alkaloid (MIA) biosynthesis have been well-known to be regulated by methyl jasmonate (MeJA)-responsive group IX ERF subfamily proteins^19,20^. However, the involvement of AP2/ERF transcription factors in the regulation of BIA biosynthesis is still unknown.

*Eschscholzia californica* (California poppy) is a traditional medicinal plant species of Native Americans and produces various BIAs that have pharmacological effects. The biosynthesis of the major metabolite sanguinarine has been intensively investigated through the characterization of biosynthesis enzyme-encoding genes. Draft genome sequencing of California poppy has been performed in recent years, and information on annotated genes is now available^21^. These useful data can enable us to search various gene families in the California poppy genome and identify candidate genes related to BIA biosynthesis.

In this study, the AP2/ERF family genes in the California poppy genome were surveyed using gene prediction data with Augustus^21^. The predicted AP2/ERF protein sequences were classified by phylogenetic analysis using the AP2/ERF domain, and gene structure, motif composition and orthologous analyses were performed using genomic and transcriptomic data. The expression patterns of the *EcAP2/ERF* genes in California poppy seedlings treated with MeJA, a key inducer of the biosynthesis of BIA as well as various plant secondary metabolites such as MIA and nicotine^19,20,22,23^, were determined by using RNA sequencing and quantitative RT-PCR (qRT-PCR) analyses. A luciferase reporter assay indicated that several *Ec*AP2/ERF proteins, which showed increased expression in response to MeJA treatment, transactivated biosynthesis enzyme gene expression. The results of this study could provide valuable information for future studies to determine the regulatory roles of AP2/ERF transcription factors in BIA biosynthesis.

## Results

### Identification of AP2/ERF transcription factor-encoding genes in the California poppy genome

We annotated 134 putative *EcAP2/ERF* genes containing AP2/ERF domain sequences from the *E. californica* draft genome database after the removal of redundant and incomplete genes. The information of individual genes is listed in Supplementary Table S1 by gene ID. Of the 134 *EcAP2/ERF* genes, *EcAP2/ERF120* was the smallest (339 bp) gene, encoding a 112 amino acid (aa) protein, while *EcAP2/ERF117* was the largest (2025 bp) gene, encoding a 674 aa protein. Among putative *Ec*AP2/ERF proteins, 15 proteins containing two AP2/ERF domains and 5 proteins containing one AP2/ERF domain together with one B3 domain were assigned to the AP2 and RAV families, respectively. The remaining proteins contained only a single AP2/ERF domain.

### Multiple sequence alignment, phylogenetic analysis, and classification of *Ec*AP2/ERF proteins

**Figure 1:**
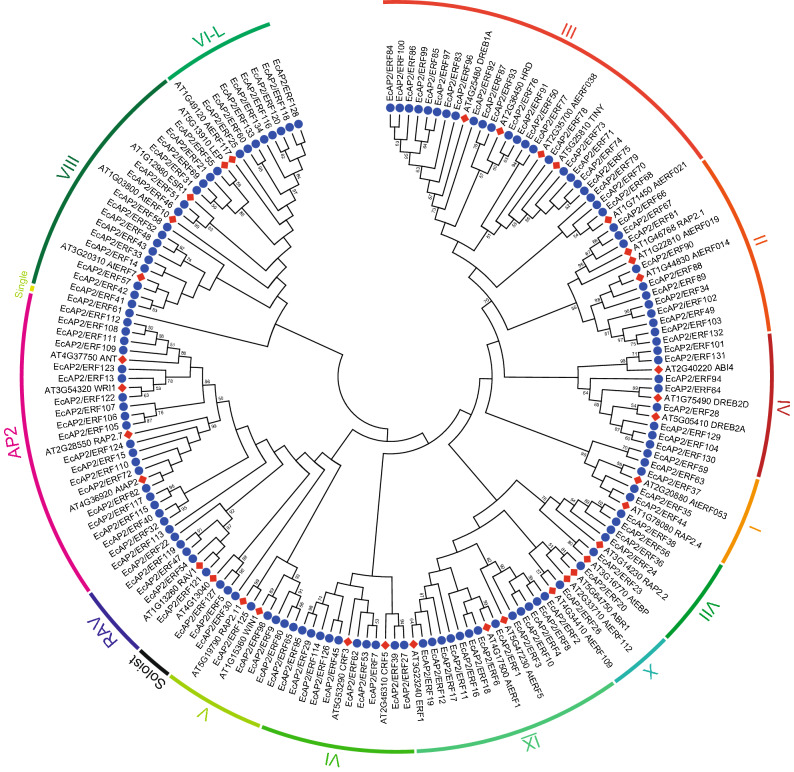
Unrooted phylogenetic tree of AP2/ERF family proteins in *E. californica* and *A. thaliana*. A NJ tree was constructed from the amino acid sequences of the AP2/ERF domain in 134 *Ec*AP2/ERF (blue circle) and 34 Arabidopsis AP2/ERF (red diamond) proteins with ID numbers using MEGA 7.0 software. Bootstrap confidence values from 1000 replicates are indicated at each branch. Each locus tag of Arabidopsis AP2/ERF protein is also described.

**Figure 2:**
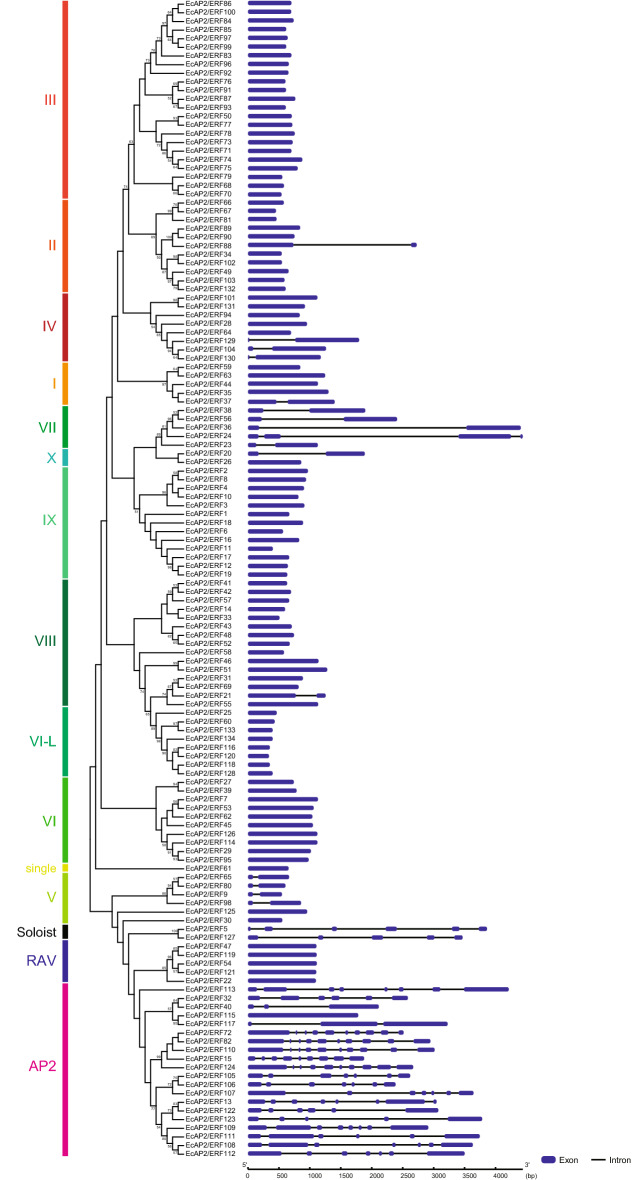
Exon–intron structure of *EcAP2/ERF* genes. A phylogenetic tree was constructed as shown in Fig. 1 using the AP2/ERF domain sequences of *Ec*AP2/ERF proteins. The gene structure of the *EcAP2/ERF* genes was visualized by the GSDS online tool. The blue boxes and black lines represent exons and introns, respectively.

To classify the 134 *Ec*AP2/ERF proteins, we performed a multiple sequence alignment of the AP2/ERF domain of the 134 *Ec*AP2/ERF proteins and of each group of *A. thaliana* AP2/ERF proteins and then constructed a phylogenetic tree using the neighbour-joining (NJ) method (Fig. 1, Supplementary Fig. S1). According to the phylogenetic tree and homology search, 5 proteins containing one AP2/ERF domain were closely related to AP2 family proteins. Thus, 20 proteins were ultimately assigned to the AP2 family. Five B3 domain-containing proteins were classified in the RAV family; however, 47 proteins were identified in the DREB subfamily, and 60 proteins belonged to the ERF subfamily. *Ec*AP2/ERF5 and *Ec*AP2/ERF127 showed homology with AT4G13040, which is involved in salicylic acid (SA)-mediated plant defence^24^; therefore, they were classified as members of the Soloist family. The DREB and ERF subfamilies were further divided into eleven groups, groups I-X and VI-L^16^. The numbers of proteins in these groups are listed in Table 1. No protein belonging to the Xb-L group was found in *E. californica*. *Ec*AP2/ERF61, which is located near group VIII, was assigned to a single group based on the phylogenetic tree, which is shown in Fig. 2. Compared with the number of AP2/ERF subfamilies in *A. thaliana* and *Vitis vinifera*, the *E. californica* genome has a similar number of DREB and ERF subfamily genes (Table 1).

**Table 1:** Summary of the numbers within each group of the AP2/ERF superfamily in *E. californica*, *A. thaliana* and *V. vinifera*.

Family	Subfamily	Group	*E. californica*	*A. thaliana*	*V. vinifera*
AP2			20	18	20
ERF			107	122	122
	DREB		47	57	40
		I	5	10	5
		II	11	15	8
		III	23	23	22
		IV	8	9	5
	ERF		60	65	82
		V	6	5	11
		VI	10	8	5
		VII	5	5	3
		VIII	15	15	11
		IX	13	17	40
		X	2	8	10
		VI-L	8	4	2
		Xb-L	0	3	0
		Single	1	0	0
RAV			5	6	6
Soloist			2	1	1
Total			134	147	149

### Gene structure and motif composition of the EcAP2/ERF family

To compare the genomic DNA sequences of *EcAP2/ERF* genes, we determined their intron and exon structures (Fig. 2). Among the 134 genes, 37 had introns, 19 AP2 family genes (except for *EcAP2/ERF115*) had at least two exons, the Soloist genes had 4–5 introns, and 16 genes in DREB and ERF subfamilies had 1–5 introns. Most of the ERF subfamily genes (85.0%) were, however, intronless. Particularly, all genes in the group III, VI, VI-L and IX subfamilies had no introns, whereas all group VII genes had more than one intron. This exon–intron structural pattern is highly consistent with that of other plant species, such as castor bean, *Brachypodium distachyon* and moso bamboo^25–27^.

**Figure 3:**
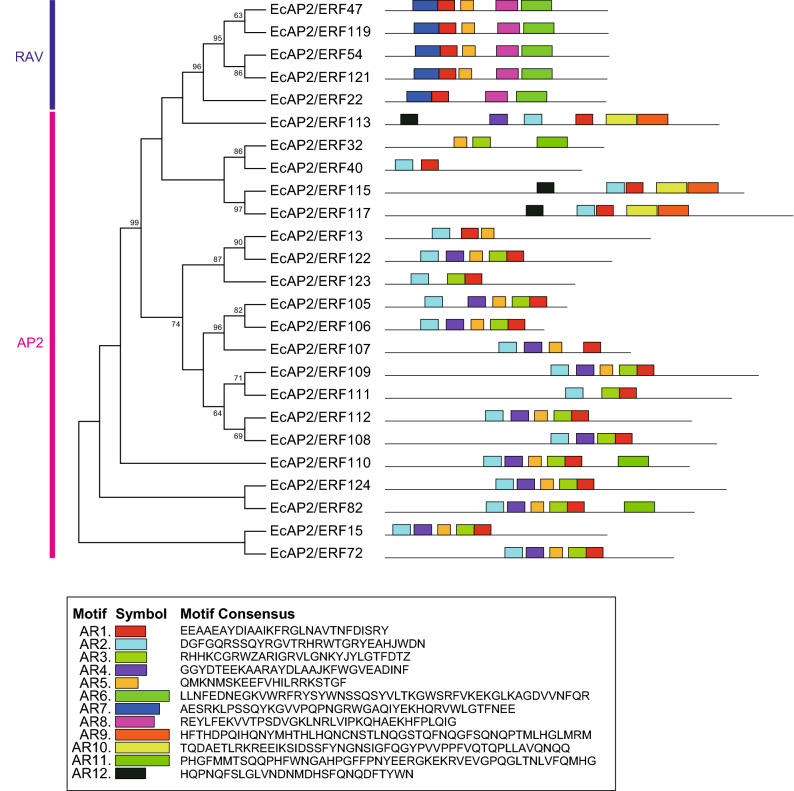
Conserved motifs of the AP2 and RAV family *Ec*AP2/ERF proteins. Twelve motifs (motifs AR1-12) were identified using the online tool MEME and are indicated by coloured rectangles. The height of the rectangles is proportional to the − log(*p*-value), truncated at the height for a motif with a *p*-value of 1e-10.

**Figure 4:**
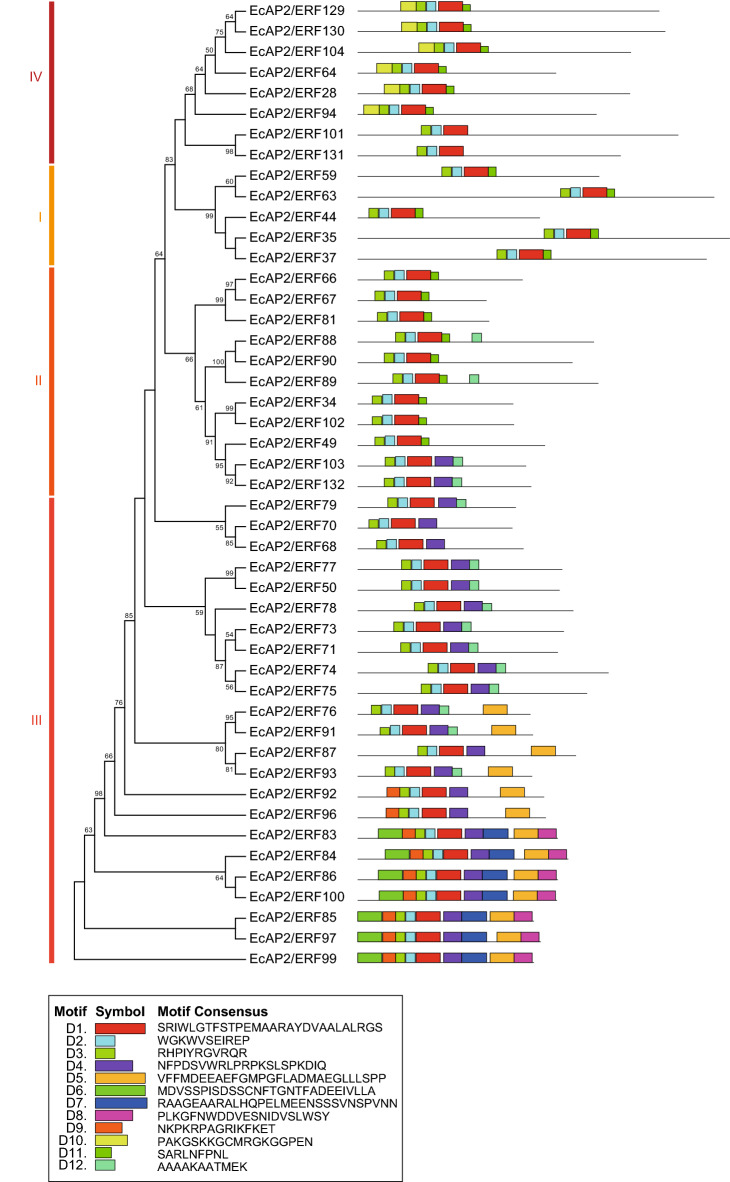
Conserved motifs of the DREB subfamily *Ec*AP2/ERF proteins. Twelve motifs (motif D1-12) were identified using the online tool MEME and are indicated by coloured rectangles.

**Figure 5:**
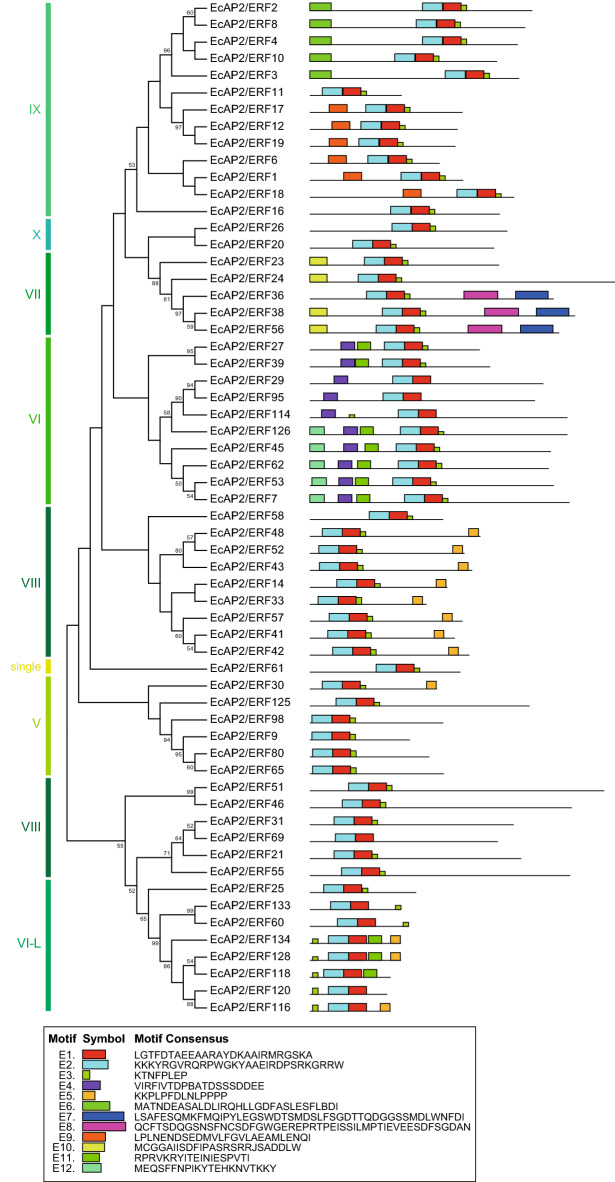
Conserved motifs of the ERF subfamily *Ec*AP2/ERF proteins. Twelve motifs (motif E1-12) were identified using the online tool MEME and are indicated by coloured rectangles.

To investigate the potential motifs of AP2/ERF proteins in each family, we analysed their amino acid sequences using online MEME analysis (Figs. 3, 4, 5). Motifs related to the AP2/ERF domain (AR1, AR2, AR3, AR4 or AR7) were found in all AP2 and RAV family proteins (Fig. 3). AR6 and AR8, which are components of the B3 domain, were found only in RAV family proteins. Several AP2 family proteins had additional motifs, AR9, AR10, AR11 and AR12. As shown in Fig. 4, all DREB subfamily (groups I-IV) proteins had D1, D2 and D3 motifs, which are related to the AP2/ERF domain. All group III proteins and two group II proteins (*Ec*AP2/ERF103 and *Ec*AP2/ERF132) had motif D4 located in the C-terminal region of the AP2/ERF domain. Some group III proteins contained D5, D6, D7, D8 and D9 motifs in the N-terminal region or C-terminal region, whereas motif D11 was found in group I, II and IV proteins. Furthermore, motif D10 was located in the N-terminal region of group IV proteins, and motif D12 was found in a portion of group II and III proteins.

Similar to the DREB subfamily proteins, all the ERF subfamily proteins (V-X, VI-like) had E1 and E2 motifs, which correspond to the AP2/ERF domain (Fig. 5). Motifs E4 and E12 were found only in group VI proteins, while motifs E7, E8 and E10 were found only in group VII proteins. Motifs E6 and E9 were found only in group IX proteins. Moreover, motif E5 was found in a portion of group V, VIII and VI-L proteins, and motif E11 was found in a portion of group VI and VI-L proteins (Fig. 5). These motifs might be important for the function of each group of proteins.

### Orthologous relationships of *Ec*AP2/ERFs between *E. californica* and *A. thaliana*

To investigate the orthologous relationship of *Ec*AP2/ERFs, we carried out a reciprocal BLAST search using predicted full-length amino acid sequences of the California poppy and Arabidopsis AP2/ERF proteins as queries. A total of 49 *Ec*AP2/ERFs reciprocally hit Arabidopsis proteins with ≥ 45% sequence similarity and ≤ 1e−20 e-value (Supplementary Table S2). Additionally, a phylogenetic tree using full-length *Ec*AP2/ERF and *At*AP2/ERF proteins was generated to validate the orthologous relationship (Supplementary Fig. S2). As a result, 19 *Ec*AP2/ERF proteins were overlapped with the reciprocal best hit proteins and 3 additional *Ec*AP2/ERFs were also predicted as orthologous proteins. These orthologous AP2/ERF proteins between *E. californica* and *A. thaliana* might likely have a similar function.

### Transcriptome-based expression profiling of *EcAP2/ERF* genes in MeJA-treated *E. californica* seedlings

**Figure 6:**
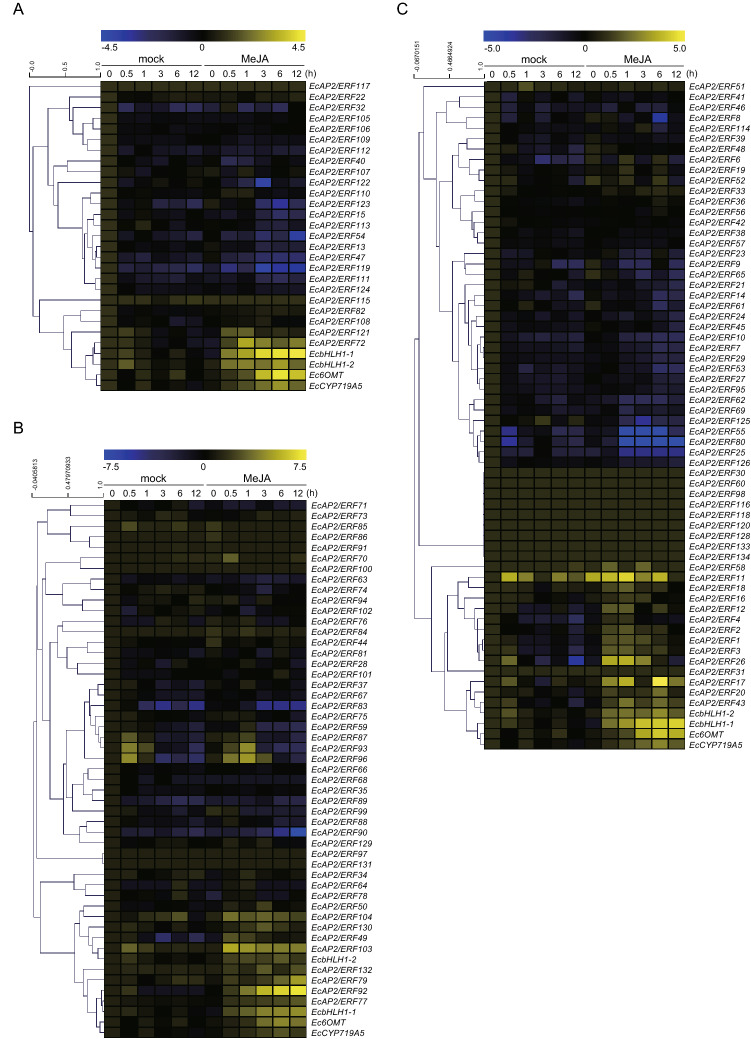
Expression profiles of *EcAP2/ERF* genes after MeJA treatment using RNA-seq data. Heat maps showing the clustering of AP2 and RAV family *EcAP2/ERF* genes (**A**), DREB subfamily *EcAP2/ERF* genes (**B**) and ERF subfamily *EcAP2/ERF* genes (**C**) with *EcbHLH1-1*, *EcbHLH1-2*, *Ec6OMT* and *EcCYP719A5* were created with log2-based FPKM values using MeV software. Within each row, the blue and yellow colours correspond to low and high values, respectively. The scale represents the signal intensity of the FPKM values.

Alkaloid production has been well known to be induced by MeJA, a phytohormone related to the defence response^28,29^. In fact, many studies have revealed that the expression of genes involved in BIA biosynthesis, including within California poppy, is transiently induced in response to MeJA^8,22,23,30^. To explore the *Ec*AP2/ERF transcription factors involved in the regulation of BIA biosynthesis, the expression profiles of *EcAP2/ERF*, BIA transcription factor-encoding genes (*EcbHLH1-1* and *EcbHLH1-2*) and biosynthesis enzyme-encoding genes (*Ec6OMT* and *EcCYP719A5*) in response to MeJA treatment were examined using RNA-seq data (Fig. 6).

As shown in Fig. 6A, the expression of *EcAP2/ERF72* in the AP2 family and *EcAP2/ERF121* in the RAV family increased in response to MeJA; the change was greater than onefold after the data were log_2_ transformed compared to the data of the mock control at 0 h. The expression of *EcbHLH1-1*, *EcbHLH1-2*, *Ec6OMT* and *EcCYP719A5* showed a clear increase after treatment with MeJA, as previously reported^8,23^. Interestingly, the expression levels of many genes in the AP2 and RAV families were downregulated under MeJA conditions.

In the DREB subfamily, more than 20 genes were highly induced by MeJA; however, many of them were also induced under the mock conditions. The expression of *EcAP2/ERF77*, *EcAP2/ERF79* and *EcAP2/ERF92* in group III was clearly upregulated by MeJA; in particular, *EcAP2/ERF92* showed the highest expression increase (fold-change > 7) after 12 h (Fig. 6B). *EcAP2/ERF49* in group II and *EcAP2/ERF130* in group IV also showed high expression increases (fold-change > 1). The expression levels of *EcAP2/ERF50*, *EcAP2/ERF103* and *EcAP2/ERF132* also increased in response to MeJA; however, their basal expression levels in California poppy seedlings were low.

In the ERF subfamily, 9 genes in group IX were highly induced (fold-change > 1) during 0.5–6 h (Fig. 6C). In particular, *EcAP2/ERF17* showed a more than approximately fivefold increase in expression after 6 h. On the other hand, the expression levels of *EcAP2/ERF8* and *EcAP2/ERF10* in group IX decrease in response to MeJA. The expression levels of *EcAP2/ERF20* and *EcAP2/ERF26* in group X and *EcAP2/ERF43* in group VIII were also apparently induced by MeJA. However, the expression profiles of the *EcAP2/ERF* genes in groups V, VI, VII and VI-L were not largely altered in response to MeJA.

### qRT-PCR analysis of *EcAP2/ERF* gene expression in response to MeJA

**Figure 7:**
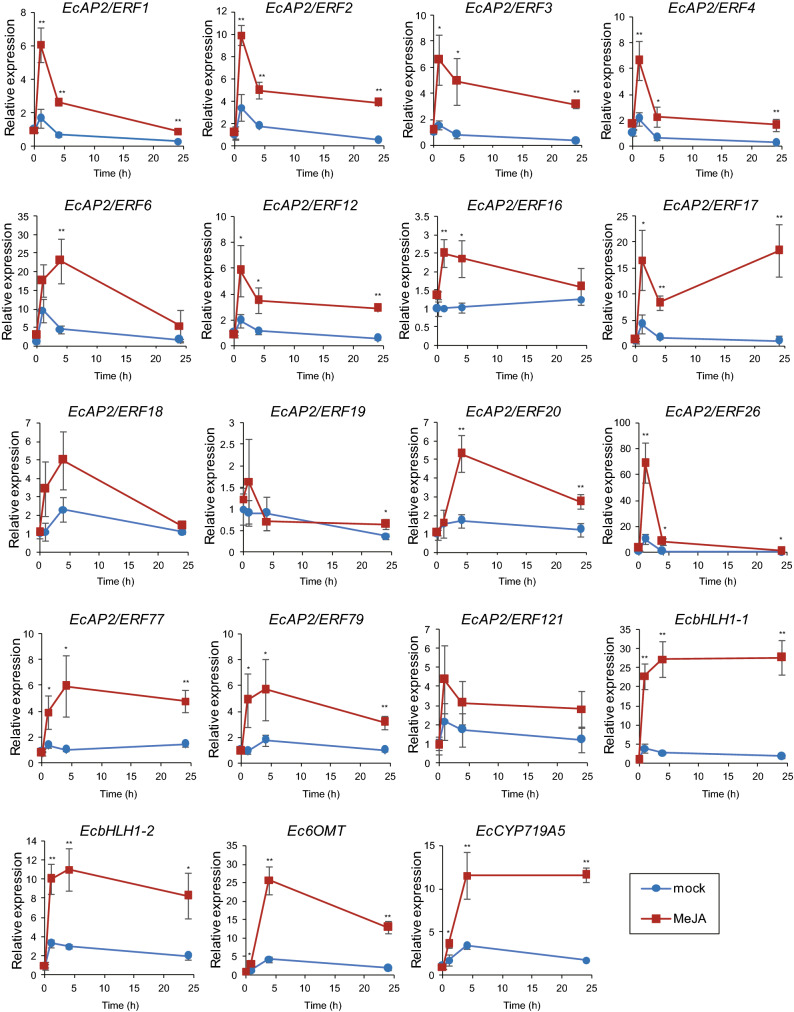
Expression levels of selected *EcAP2/ERF* genes in California poppy seedlings treated with 100 µM MeJA. The transcript levels of 15 *EcAP2/ERF* genes and *EcbHLH1-1*, *EcbHLH1-2*, *Ec6OMT* and *EcCYP719A5* were determined by qRT-PCR. The relative expression levels represent the values standardized by those of the mock 0 h samples, which were set to 1. The error bars indicate the SDs calculated from three biological replicates. The asterisks denote significant differences according to Student's *t*-test compared with the mocks: *P < 0.05; **P < 0.01.

To further verify the expression levels of the *EcAP2/ERF* genes that showed an increase in response to MeJA via RNA sequencing, qRT-PCR analysis was performed using cDNA derived from 6 MeJA-treated seedlings, with three biological replications. We selected 15 *EcAP2/ERF* genes for qRT-PCR analysis because the basal expression levels of several genes, such as *EcAP2/ERF92* and *EcAP2/ERF103*, were too low or because the genes were not stably expressed in California poppy seedlings. Four BIA biosynthesis genes, *EcbHLH1-1*, *EcbHLH1-2*, *Ec6OMT* and *EcCYP719A5*, were also analysed, as shown in Fig. 6, and a clear response to MeJA was confirmed, as previously described. As shown in Fig. 7, the expression of group IX *EcAP2/ERF* genes (except for *EcAP2/ERF19*) was highly upregulated in response to MeJA. The expression patterns of *EcAP2/ERF1*, *EcAP2/ERF2*, *EcAP2/ERF3*, *EcAP2/ERF4*, *EcAP2/ERF12* and *EcAP2/ERF16* were very similar in that the highest expression was shown at 1 h; however, the expression of *EcAP2/ERF6* and *EcAP2/ERF18* was the highest at 4 h, and that of *EcAP2/ERF17* was the highest at 24 h. The expression patterns of *EcAP2/ERF77* and *EcAP2/ERF79*, which are group III DREB genes, were also similar. Two group X genes, *EcAP2/ERF20* and *EcAP2/ERF26*, showed clear MeJA responsiveness, though their expression patterns were slightly different. In particular, the expression of *EcAP2/ERF26* showed a more than 70-fold increase at 1 h compared to that of the mock controls. The expression of *EcAP2/ERF121*, which are RAV family genes, increased in response to MeJA, but a significant difference was not observed because of the large variation in the data. These qRT-PCR analysis results supported the MeJA-responsive expression of several *EcAP2/ERF* genes revealed by the RNA sequencing analysis.

### Transactivation activity of EcAP2/ERF proteins

**Figure 8:**
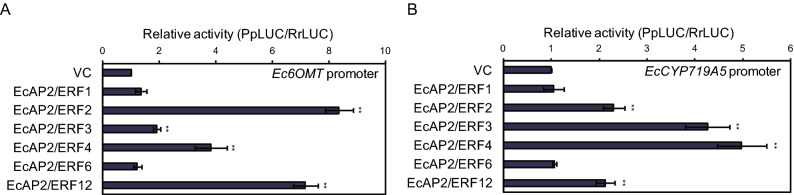
Transcriptional activity of group IX *Ec*AP2/ERF proteins. Transactivation of the *Ec6OMT* (**A**) and *EcCYP719A5* (**B**) promoter::*LUC* reporter genes by several group IX *Ec*AP2/ERF proteins. LUC activities were determined by a dual-LUC reporter assay. The relative activity was standardized by using the value of VC as 1. The error bars indicate the SDs calculated from three biological transfections. The asterisks denote significant differences according to Student's *t-*test compared with the VC: **P < 0.01.

Since expression analysis indicated that many group IX *EcAP2/ERF* genes have MeJA responsiveness, we further investigated the role of these *Ec*AP2/ERF proteins in the transactivation of BIA biosynthesis enzyme-encoding genes via a transient luciferase (LUC) reporter assay (Fig. 8). Relative LUC activity produced by *Ec6OMT* and *EcCYP719A5* gene promoter::*LUC* constructs was significantly induced by *Ec*AP2/ERF2, *Ec*AP2/ERF3, *Ec*AP2/ERF4 and *Ec*AP2/ERF12, suggesting that these *Ec*AP2/ERF transcription factors function as transcriptional activators during BIA biosynthesis.

## Discussion

The AP2/ERF superfamily is one of the largest groups of plant-specific transcription factors involved in plant developmental processes as well as biotic and/or abiotic stress response and various plant hormone signalling^18^. Members of the AP2/ERF superfamily have been studied at genome-wide level in various plant species, such as Arabidopsis, soybean, rice, carrot, moso bamboo, Chinese jujube and grape^15,26,31–35^. However, this study is the first report on the genome-wide identification of AP2/ERF transcription factor-encoding genes from *E. californica*, a BIA-producing plant. In this study, 134 AP2/ERF members were identified from the California poppy genome (Table 1), whose size was 502 Mb^36^. The number of *AP2/ERF* genes in California poppy was quite similar to that in *A. thaliana* (125 Mb), *V. vinifera* (490 Mb), and *Oryza sativa* L. *japonica* (389 Mb), with 147, 149 and 163 AP2/ERF family genes, respectively^16,32,33^. On the other hand, poplar (485 Mb), Chinese cabbage (485 Mb), and carrot (480 Mb) have more than 200 AP2/ERF family genes^34,37,38^. Furthermore, a total of 52 *Ec*AP2/ERF proteins showed predicted orthologous relationship with Arabidopsis AP2/ERF proteins (Supplementary Fig. S2 and Table S2). These findings indicate that the numbers of AP2/ERF transcription factor-encoding genes vary among plant species, and their functions have were expanded throughout evolution. The genome of California poppy, a basal eudicot member of the Papaveraceae family, may indicate the evolutionary history of AP2/ERF family genes in land plants. As shown by the summary of the number and gene density (per megabase) of AP2/ERF family genes from various plant species (Supplementary Table S3), the percentage of each family gene in *E. californica* is relatively close to that in other plant species. The gene density of *E. californica*, 0.267, is close to that of *Medicago truncatula* and is relatively low compared to that of other plant species. Since several *EcAP2/ERF* genes containing incomplete AP2/ERF domain sequences were omitted in this study, however, the exact number of *AP2/ERF* genes in the California poppy genome may be higher.

Gene structural analysis showed that 72.4% of *EcAP2/ERF* genes had no introns, while the AP2 subfamily genes exhibited complex structures; that is, nearly all AP2 family genes in California poppy harboured introns ranging from 1 to 9 (Fig. 2). This tendency is consistent with phenomena observed in other plant species^25–27^. The conserved exon–intron structure of AP2 family genes suggests that gene duplication might have occurred after the evolution of introns, and the small number of AP2 family genes might be due to the intron structure, which impairs the ability to duplicate. On the other hand, the lack of introns in many DREB and ERF subfamily genes might suggest the need for these genes for a quick response to environmental stresses during development.

Conserved domains and motifs of transcription factors are generally important for their transcriptional activity, protein–protein interactions and DNA-binding activity. Motif analysis revealed that each family and subgroup of proteins shared similar motifs, which might be related to their specific functions (Figs. 3, 4, 5). It is also possible to speculate about the function of *Ec*AP2/ERF proteins compared with those of the motifs of Arabidopsis and rice AP2/ERF proteins^16^. Since motif D9 was found in only subgroup IIIc proteins, it might confer a specific function to those proteins. The D8 motif found in a subset of group III proteins contains an LWSY sequence at the C-terminus, which is also conserved in *Os*DREB1A, B and C and in *At*DREB1A as an LWSY motif^16^. These proteins have been reported to be involved in high salt, drought and cold tolerance^39^. Further, a DSVWR sequence in motif D4, which is similar to the conserved DSAWR sequence found in Arabidopsis DREB proteins, is present in group II and III proteins; however, the function of this motif is still unknown. Motif E5 contains an FDLNxxP sequence, which is well known as an EAR motif^40^. Since the EAR motif affords repression activity, *Ec*AP2/ERF proteins that harbour E5 motifs might function as transcriptional repressors. Motifs E4, E11 and E12 were found mainly in group VI proteins, which suggests that these motifs might exhibit characteristic features of group VI proteins. Motif E8 is conserved in group VII proteins and contains a TPEISS sequence, which is considered a putative MAP kinase phosphorylation site^16^. Thus, the function of group VII proteins containing the E8 motif might be regulated by MAP kinase-related signalling. Motifs E6 and E9 are present in group IX proteins, which can be divided into three subgroups, IXa, IXb and IXc^16^. Motif E6 is present in subgroup IXb proteins, while motif E9 is present in subgroup IXa and IXc proteins. It is possible that these motifs might provide specific functions of each subgroup of proteins and might be related to the transactivation activity of group IX proteins, as shown in Fig. 8. Motifs AR1, AR2, AR3, AR4 and AR7 in AP2 and RAV family proteins are included in AP2/ERF domain sequences. The other motifs, such as AR5, AR9, AR10, AR11 and AR12, contain unique sequences that might confer specific functions to these proteins.

Plant hormone signaling has an important influence on various plant processes including the biosynthesis of secondary metabolites^28,29^. Whereas alkaloid biosynthesis has been also reported to be affected by various plant hormones^2,41,42^, BIA biosynthesis is quite well-known to be controlled by JA-signalling involved in chemical defence against microorganisms and herbivores in the plants, including California poppy^8,22,23^. Potential AP2/ERF transcription factors involved in the regulation of BIA biosynthesis in California poppy were characterized by analysis of their gene expression profiles via RNA sequencing and qRT-PCR based on MeJA-responsiveness. The results revealed that 14 *EcAP2/ERF* genes were upregulated in response to MeJA, though 2 genes showed no significant difference (Figs. 6 and 7). In particular, the expression of 9 of 13 group IX *EcAP2/ERF* genes clearly increased in response to MeJA within 4 h. Compared to the expression pattern of *Ec6OMT* and *EcCYP719A5*, the expression pattern of many group IX genes was upregulated rapidly more but then decreased. This quick MeJA response is relatively similar to the response of *EcbHLH1-1* and EcbHLH1-2, both of which are potential master regulators in BIA biosynthesis.

Group IX AP2/ERF transcription factors have been reported to be involved in the regulation of various secondary metabolic pathways^20^. Octadecanoid derivative-responsive *Catharanthus* AP2-domain (ORCA) 2 and 3 were first isolated from *Catharanthus roseus* as MeJA-responsive group IX AP2/ERF transcriptional regulators of alkaloid biosynthesis^43,44^. In particular, ORCA3 is considered a master regulator of monoterpenoid indole alkaloid (MIA) biosynthesis^45^. In addition, the following proteins have been identified and characterized in the last decade: *Nt*ERF189, a master regulator of nicotine biosynthesis in *Nicotiana tabacum*^46^; *Aa*ERF1, *Aa*ERF2 and *Aa*ORA, positive regulators of artemisinin biosynthesis in *Artemisia annua*^47,48^; GAME9/JRE4, a master regulator of steroidal glycoalkaloid biosynthesis in tomato and potato^49,50^; and *Op*ERF2, a possible regulator of camptothecin biosynthesis in *Ophiorrhiza pumila*^51^. These group IX proteins showed high similarity. These findings strongly suggest that MeJA-responsive group IX *Ec*AP2/ERF transcription factors are involved in the positive regulation of BIA biosynthesis. In fact, in the present study, a transient LUC reporter assay indicated that four group IX *Ec*AP2/ERF proteins were clearly upregulated, reflecting transcriptional activity within the promoters of biosynthesis genes (Fig. 8).

Interestingly, tobacco group IX *AP2/ERF* genes, including *NtERF189*, are known to be clustered in the nicotine production regulatory locus called *NIC2*^46^. Furthermore, *ORCA* gene clusters were identified from the *C. roseus* genomic scaffold^52,53^. A recent study revealed that these AP2/ERF transcription factors encoded by the *ORCA* gene cluster mutually regulate the expression of clustered genes and MIA biosynthesis^54^. To examine whether *EcAP2/ERF* genes were also clustered in the California poppy genome, we searched for the location of each *EcAP2/ERF* gene in the genome scaffolds. The results revealed that the *EcAP2/ERF2*, *EcAP2/ERF3* and *EcAP2/ERF4* genes, which showed high upregulation in response to MeJA, co-localized in the same genome scaffold (Supplementary Table S4). These *Ec*AP2/ERF proteins showed the highest similarity to Arabidopsis *At*ERF5 in the orthologous analysis (Supplementary Table S2). Further, *Ec*AP2/ERF2, *Ec*AP2/ERF3 and *Ec*AP2/ERF4 exhibited relatively high transactivation activity in the reporter assay. Our findings strongly suggest that these clustered *EcAP2/ERF* genes might be derived from common origin through gene duplication and these AP2/ERF transcription factors play an important role in the regulation of BIA biosynthesis. Additional functional characterization of *Ec*AP2/ERF transcription factors and elucidation of the transcriptional network involved in several transcription factors, including AP2/ERF proteins, are ongoing.

## Materials and methods

### Identification of AP2/ERF superfamily genes in the *E. californica* draft genome

Based on the annotated gene information available at the Eschscholzia Genome DataBase (https://eschscholzia.kazusa.or.jp), 178 *AP2/ERF* genes were first isolated from the California poppy draft genome. After removing redundant and incomplete ORF sequences, the SMART database (https://smart.embl-heidelberf.de/) was used to further eliminate sequences that did not contain a complete AP2/ERF domain. All the genes were also validated using the PhytoMetaSyn transcriptomic database (https://bioinformatics.tugraz.at/phytometasyn/)^55^ and the NCBI database (https://www.ncbi.nlm.nih.gov/) by BLAST (Supplementary Table S5). Since the *EcAP2/ERF2* and *EcAP2/ERF44* genes showed clear differences in CDS length between the Eschscholzia Genome DataBase and PhytoMetaSyn, the precise length of these genes was defined by isolation and sequencing. In total, 134 *EcAP2/ERF* genes were ultimately identified in the *E. californica* draft genome and defined for further analyses.

### Alignment and phylogenetic analyses

Multiple sequence alignment of AP2/ERF domain sequences in *A. thaliana* and *E. californica* AP2/ERF transcription factors by ClustalW was visualized using BioEdit software (https://www.mbio.ncsu.edu/BioEdit/bioedit.html). An unrooted phylogenetic tree was generated with the NJ method, Jones-Thornton-Taylor (JTT) model, and 1000 bootstrap replications using MEGA 7.0 software (https://www.megasoftware.net/)^56^.

### Gene structure and conserved motif analysis

Gene structure was visualized using the online Gene Structure Display Server (GSDS) (https://gsds.cbi.pku.edu.cn/) based on predicted full-length CDS regions with their corresponding genomic sequences. Conserved motifs of AP2 and RAV family members and DREB and ERF subfamily proteins were predicted using the MEME Suite web server version 5.1.0 (https://meme-suite.org/) with the following parameters: maximum number of motifs of 12 and optimum motif width ≥ 6 and ≤ 50^57^. The topology of the phylogenetic trees was determined with the AP2/ERF domain using MEGA 7.0 software.

### Orthologous analysis

A reciprocal BLAST was carried out to find the orthologous relationship between California poppy and Arabidopsis AP2/ERF proteins. Best hits with more than 50% sequence coverage were picked up. Un-rooted phylogenetic tree using full-length *Ec*AP2/ERF and *At*AP2/ERF proteins was generated with the NJ method, p-distance model, and 1000 bootstrap replications using MEGA7.0.

### RNA sequencing and expression profiling

California poppy seedlings were grown in Linsmaier-Skoog (LS) media (pH 5.7, 0.8% agar) at 23 °C under continuous light (50–100 µmol photons/m^2^/s) for 10 days, after which they were transferred to LS media supplemented with 100 µM MeJA or 0.1% dimethylsulfoxide (DMSO) as a control. Total RNA was extracted from 5 seedlings at 0, 0.5, 1, 3, 6 and 12 h after treatment using an RNeasy Plant Mini Kit (Qiagen, Hilden, Germany) and subsequently sequenced as 101 bp + 101 bp paired-ends on an Illumina HiSeq 2500 by Hokkaido System Science Co., Ltd. (Hokkaido, Japan). Read trimming using the cutadapt and Trimmomatic programs, read mapping using the TopHat/Bowtie program, and expression analysis via calculations of fragments per kilobase of exon model per million mapped fragments (FPKM) values using Cufflinks were also performed. Hierarchical clustering and construction of heat maps were performed using MeV software (https://mev.tm4.org/).

### Gene expression analysis by real-time PCR

Eighteen seedlings of California poppy that were grown as described above were treated with 100 µM MeJA for 0, 1, 4 and 24 h. After dividing them into three sample groups (each with 6 seedlings), total RNA was extracted from each group using an RNeasy Plant Mini Kit. Single-stranded cDNA was synthesized from 1 µg of total RNA using ReverTra Ace qPCR RT Master Mix in conjunction with gDNA Remover (Toyobo, Osaka, Japan). Quantitative RT-PCR was performed with specific primer pairs (Supplementary Table S6) using Thunderbird SYBR qPCR Mix (Toyobo, Osaka, Japan) and a LightCycler 96 system (Roche, Basel, Switzerland). The PCR conditions were 95 °C for 1 min, followed by 45 cycles of 95 °C for 15 s, 60 °C for 20 s and 72 °C for 30 s. The data were calculated by the comparative 2^−ΔΔCt^ method, and the relative expression levels were standardized by the amplification of the *β-actin* gene as an internal control.

### LUC reporter assay

The promoter sequences of *Ec6OMT* (507 bp) and *EcCYP719A5* (621 bp) were isolated from California poppy genomic DNA and inserted into a pDR196-*LUC* vector, yielding a promoter::*LUC* reporter construct. The full-length cDNAs of group IX *EcAP2/ERF* genes were fused to the CaMV 35S promoter in the pBI221 vector, which subsequently served as an effector construct. A dual-LUC reporter assay was then performed using *C. japonica* protoplasts as previously described^58^.

## Supplementary information

Supplementary Information.
