# Supplementary material for: Genome-wide identification of AP2/ERF transcription factor-encoding genes in California poppy (Eschscholzia californica) and their expression profiles in response to methyl jasmonate
Source: Sci Rep. 2020 Oct 22;10:18066. doi: 10.1038/s41598-020-75069-7 (PMC7582171; doi:10.1038/s41598-020-75069-7)
Supplement: Supplementary file 1 — Supplementary Information. [file 41598_2020_75069_MOESM1_ESM.pdf]

Supplementary information

Genome-wide identification of AP2/ERF transcription factor-encoding genes in California poppy (*Eschscholzia californica*) and their expression profiles in response to methyl jasmonate

Yasuyuki Yamada[1,*], Shohei Nishida[2], Nobukazu Shitan[1], and Fumihiko Sato[2,3,*]

[1]Laboratory of Medicinal Cell Biology, Kobe Pharmaceutical University, Kobe, Japan

[2]Department of Plant Gene and Totipotency, Division of Integrated Life Science, Graduate School of Biostudies, Kyoto University, Kyoto, Japan

[3]Graduate School of Science, Osaka Prefecture University, Sakai, Japan

[*]Corresponding authors

Email: yyamada@kobepharma-u.ac.jp/fsato@lif.kyoto-u.ac.jp

Supplementary Table S1

Identification of AP2/ERF transcription factor gene family in *E. californica*.

Name	Gene ID	CDS length	Genome length	Protein length	Subfamily	Subgroup
EcAP2/ERF1	Eca_sc000448.1_g0520.1	669	669	222	ERF	IX
EcAP2/ERF2	Eca_sc006292.1_g0200.1	969	969	322	ERF	IX
EcAP2/ERF3	Eca_sc006292.1_g0150.1	912	912	303	ERF	IX
EcAP2/ERF4	Eca_sc006292.1_g0190.1	906	906	301	ERF	IX
EcAP2/ERF5	Eca_sc194689.1_g0210.1	693	3862	230	Soloist	
EcAP2/ERF6	Eca_sc000360.1_g1660.1	567	567	188	ERF	IX
EcAP2/ERF7	Eca_sc000360.1_g0090.1	1131	1131	376	ERF	VI
EcAP2/ERF8	Eca_sc003794.1_g0010.1	939	939	312	ERF	IX
EcAP2/ERF9	Eca_sc194674.1_g0040.1	438	549	145	ERF	V
EcAP2/ERF10	Eca_sc001253.1_g1650.1	816	816	271	ERF	IX
EcAP2/ERF11	Eca_sc194641.1_g1360.1	402	402	133	ERF	IX
EcAP2/ERF12	Eca_sc194641.1_g1370.1	645	645	214	ERF	IX
EcAP2/ERF13	Eca_sc194697.1_g0440.1	1320	3048	439	AP2-like	
EcAP2/ERF14	Eca_sc194641.1_g0520.1	600	600	199	ERF	VIII
EcAP2/ERF15	Eca_sc186325.1_g1660.1	1104	1877	367	AP2	
EcAP2/ERF16	Eca_sc183659.1_g1650.1	828	828	275	ERF	IX
EcAP2/ERF17	Eca_sc194316.1_g0160.1	666	666	221	ERF	IX
EcAP2/ERF18	Eca_sc004486.1_g0660.1	891	891	296	ERF	IX
EcAP2/ERF19	Eca_sc003030.1_g0120.1	636	636	211	ERF	IX
EcAP2/ERF20	Eca_sc194486.1_g2890.1	804	1888	267	ERF	X
EcAP2/ERF21	Eca_sc194486.1_g2620.1	921	1255	306	ERF	VIII
EcAP2/ERF22	Eca_sc194486.1_g3490.1	1098	1098	365	RAV	
EcAP2/ERF23	Eca_sc194094.1_g0110.1	825	1129	274	ERF	VII
EcAP2/ERF24	Eca_sc194094.1_g0160.1	1329	4439	442	ERF	VII
EcAP2/ERF25	Eca_sc000145.1_g0920.1	465	465	154	ERF	VI-L
EcAP2/ERF26	Eca_sc001928.1_g3020.1	861	861	286	ERF	X
EcAP2/ERF27	Eca_sc001928.1_g2560.1	741	741	246	ERF	VI
EcAP2/ERF28	Eca_sc001928.1_g2870.1	954	954	317	DREB	IV
EcAP2/ERF29	Eca_sc194692.1_g0080.1	1017	1017	338	ERF	VI
EcAP2/ERF30	Eca_sc014577.1_g1290.1	555	555	184	ERF	V
EcAP2/ERF31	Eca_sc194732.1_g0950.1	888	888	295	ERF	VIII
EcAP2/ERF32	Eca_sc194794.1_g0100.1	1089	2584	362	AP2-like	

EcAP2/ERF33	Eca_sc184725.1_g0010.1	510	510	169	ERF	VIII
EcAP2/ERF34	Eca_sc011255.1_g1900.1	546	546	181	DREB	II
EcAP2/ERF35	Eca_sc007884.1_g2280.1	1302	1302	433	DREB	I
EcAP2/ERF36	Eca_sc006305.1_g0100.1	1062	4408	353	ERF	VII
EcAP2/ERF37	Eca_sc000631.1_g1580.1	1221	1401	406	DREB	I
EcAP2/ERF38	Eca_sc194755.1_g0250.1	1155	1896	384	ERF	VII
EcAP2/ERF39	Eca_sc000661.1_g2160.1	786	786	261	ERF	VI
EcAP2/ERF40	Eca_sc010932.1_g0930.1	978	2114	325	AP2-like	
EcAP2/ERF41	Eca_sc003774.1_g0170.1	633	633	210	ERF	VIII
EcAP2/ERF42	Eca_sc003774.1_g0160.1	696	696	231	ERF	VIII
EcAP2/ERF43	Eca_sc002218.1_g1810.1	708	708	235	ERF	VIII
EcAP2/ERF44	Eca_sc001921.1_g0160.1	1131	1131	376	DREB	I
EcAP2/ERF45	Eca_sc000102.1_g0370.1	1050	1050	349	ERF	VI
EcAP2/ERF46	Eca_sc194696.1_g0530.1	1140	1140	379	ERF	VIII
EcAP2/ERF47	Eca_sc194696.1_g0250.1	1107	1107	368	RAV	
EcAP2/ERF48	Eca_sc194668.1_g0170.1	744	744	247	ERF	VIII
EcAP2/ERF49	Eca_sc194668.1_g0700.1	657	657	218	DREB	II
EcAP2/ERF50	Eca_sc194668.1_g0380.1	708	708	235	DREB	III
EcAP2/ERF51	Eca_sc010606.1_g0230.1	1281	1281	426	ERF	VIII
EcAP2/ERF52	Eca_sc001936.1_g0240.1	675	675	224	ERF	VIII
EcAP2/ERF53	Eca_sc001139.1_g2640.1	1062	1062	353	ERF	VI
EcAP2/ERF54	Eca_sc001139.1_g1450.1	1113	1113	370	RAV	
EcAP2/ERF55	Eca_sc194718.1_g0290.1	1134	1134	377	ERF	VIII
EcAP2/ERF56	Eca_sc003413.1_g1270.1	1086	2410	361	ERF	VII
EcAP2/ERF57	Eca_sc001904.1_g0290.1	666	666	221	ERF	VIII
EcAP2/ERF58	Eca_sc194483.1_g1620.1	582	582	193	ERF	VIII
EcAP2/ERF59	Eca_sc003893.1_g0980.1	846	846	281	DREB	I
EcAP2/ERF60	Eca_sc000537.1_g1470.1	432	432	143	ERF	VI-L
EcAP2/ERF61	Eca_sc194561.1_g0040.1	657	657	218	ERF	single
EcAP2/ERF62	Eca_sc013037.1_g0010.1	1041	1041	346	ERF	VI
EcAP2/ERF63	Eca_sc194713.1_g0160.1	1248	1248	415	DREB	I
EcAP2/ERF64	Eca_sc194610.1_g0270.1	696	696	231	DREB	IV
EcAP2/ERF65	Eca_sc000462.1_g0180.1	585	665	194	ERF	V
EcAP2/ERF66	Eca_sc194677.1_g0010.1	579	579	192	DREB	II
EcAP2/ERF67	Eca_sc000399.1_g0490.1	453	453	150	DREB	II
EcAP2/ERF68	Eca_sc194541.1_g1820.1	582	582	193	DREB	III

EcAP2/ERF69	Eca_sc194541.1_g1650.1	819	819	272	ERF	VIII
EcAP2/ERF70	Eca_sc194620.1_g0290.1	543	543	180	DREB	III
EcAP2/ERF71	Eca_sc194565.1_g0440.1	702	702	233	DREB	III
EcAP2/ERF72	Eca_sc004486.1_g0140.1	1434	2515	477	AP2	
EcAP2/ERF73	Eca_sc001713.1_g0320.1	723	723	240	DREB	III
EcAP2/ERF74	Eca_sc001301.1_g0740.1	879	879	292	DREB	III
EcAP2/ERF75	Eca_sc000630.1_g2480.1	804	804	267	DREB	III
EcAP2/ERF76	Eca_sc000630.1_g3370.1	606	606	201	DREB	III
EcAP2/ERF77	Eca_sc003926.1_g0060.1	717	717	238	DREB	III
EcAP2/ERF78	Eca_sc000587.1_g0100.1	756	756	251	DREB	III
EcAP2/ERF79	Eca_sc194602.1_g1910.1	555	555	184	DREB	III
EcAP2/ERF80	Eca_sc003743.1_g1960.1	522	606	173	ERF	V
EcAP2/ERF81	Eca_sc001754.1_g0540.1	462	462	153	DREB	II
EcAP2/ERF82	Eca_sc002478.1_g1480.1	1536	2948	511	AP2	
EcAP2/ERF83	Eca_sc000388.1_g0060.1	702	702	233	DREB	III
EcAP2/ERF84	Eca_sc000388.1_g0070.1	738	738	245	DREB	III
EcAP2/ERF85	Eca_sc000388.1_g0090.1	618	618	205	DREB	III
EcAP2/ERF86	Eca_sc000388.1_g0100.1	702	702	233	DREB	III
EcAP2/ERF87	Eca_sc001536.1_g0070.1	765	765	254	DREB	III
EcAP2/ERF88	Eca_sc194756.1_g1130.1	828	2726	275	DREB	II
EcAP2/ERF89	Eca_sc186929.1_g0010.1	843	843	280	DREB	II
EcAP2/ERF90	Eca_sc002196.1_g0380.1	753	753	250	DREB	II
EcAP2/ERF91	Eca_sc001224.1_g0560.1	615	615	204	DREB	III
EcAP2/ERF92	Eca_sc001224.1_g0630.1	654	654	217	DREB	III
EcAP2/ERF93	Eca_sc194570.1_g1150.1	612	612	203	DREB	III
EcAP2/ERF94	Eca_sc001060.1_g0470.1	837	837	278	DREB	IV
EcAP2/ERF95	Eca_sc002447.1_g0680.1	981	981	326	ERF	VI
EcAP2/ERF96	Eca_sc001096.1_g0990.1	660	660	219	DREB	III
EcAP2/ERF97	Eca_sc000108.1_g1870.1	642	642	213	DREB	III
EcAP2/ERF98	Eca_sc002391.1_g1110.1	582	858	193	ERF	V
EcAP2/ERF99	Eca_sc000108.1_g1900.1	618	618	205	DREB	III
EcAP2/ERF100	Eca_sc000108.1_g1920.1	699	699	232	DREB	III
EcAP2/ERF101	Eca_sc004328.1_g0980.1	1122	1122	373	DREB	IV
EcAP2/ERF102	Eca_sc194665.1_g0060.1	549	549	182	DREB	II
EcAP2/ERF103	Eca_sc003758.1_g2210.1	591	591	196	DREB	II
EcAP2/ERF104	Eca_sc001803.1_g0310.1	957	1262	318	DREB	IV

EcAP2/ERF105	Eca_sc002150.1_g1110.1	906	2621	301	AP2	
EcAP2/ERF106	Eca_sc002983.1_g1490.1	792	2385	263	AP2	
EcAP2/ERF107	Eca_sc002983.1_g1530.1	1221	3644	406	AP2	
EcAP2/ERF108	Eca_sc000153.1_g0130.1	1647	3633	548	AP2	
EcAP2/ERF109	Eca_sc004205.1_g0740.1	1854	2914	617	AP2	
EcAP2/ERF110	Eca_sc001539.1_g0350.1	1512	3016	503	AP2	
EcAP2/ERF111	Eca_sc005409.1_g0720.1	1722	3745	573	AP2	
EcAP2/ERF112	Eca_sc005351.1_g0210.1	1524	3501	507	AP2	
EcAP2/ERF113	Eca_sc002690.1_g1650.1	1659	4213	552	AP2	
EcAP2/ERF114	Eca_sc001479.1_g1220.1	1122	1122	373	ERF	VI
EcAP2/ERF115	Eca_sc194672.1_g1110.1	1782	1782	593	AP2-like	
EcAP2/ERF116	Eca_sc000537.1_g1530.1	354	354	117	ERF	VI-L
EcAP2/ERF117	Eca_sc000212.1_g0400.1	2025	3226	674	AP2-like	
EcAP2/ERF118	Eca_sc000537.1_g1520.1	354	354	117	ERF	VI-L
EcAP2/ERF119	Eca_sc000635.1_g0070.1	1110	1110	369	RAV	
EcAP2/ERF120	Eca_sc000537.1_g1480.1	339	339	112	ERF	VI-L
EcAP2/ERF121	Eca_sc184896.1_g0290.1	1104	1104	367	RAV	
EcAP2/ERF122	Eca_sc002317.1_g0300.1	1128	3076	375	AP2	
EcAP2/ERF123	Eca_sc003758.1_g2660.1	945	3784	314	AP2	
EcAP2/ERF124	Eca_sc000587.1_g0190.1	1695	2670	564	AP2	
EcAP2/ERF125	Eca_sc001493.1_g0570.1	957	957	318	ERF	V
EcAP2/ERF126	Eca_sc194480.1_g0330.1	1122	1122	373	ERF	VI
EcAP2/ERF127	Eca_sc194779.1_g0120.1	684	3468	227	Solist	
EcAP2/ERF128	Eca_sc000537.1_g1550.1	399	399	132	ERF	VI-L
EcAP2/ERF129	Eca_sc000537.1_g0740.1	1056	1709	351	DREB	IV
EcAP2/ERF130	Eca_sc003765.1_g1250.1	1077	1177	358	DREB	IV
EcAP2/ERF131	Eca_sc003765.1_g1130.1	921	921	306	DREB	IV
EcAP2/ERF132	Eca_sc186764.1_g0090.1	609	609	202	DREB	II
EcAP2/ERF133	Eca_sc000537.1_g1560.1	399	399	132	ERF	VI-L
EcAP2/ERF134	Eca_sc000537.1_g1460.1	399	399	132	ERF	VI-L

AP2-like has single AP2/ERF domain.

Supplementary Table S2

The orthologous relationship between *Ec*AP2/ERFs and *At*AP2/ERFs.

EcAP2/ERFs	Gene ID of AtAP2/ERFs		Identities (%)	Positives (%)	Score (bits)	E-value	Reciprocal Best Hit	Phylogenetic relationship
EcAP2/ERF1	AT4G17500	AtERF1	57	68	206	3.00E-56	○	
EcAP2/ERF2	AT5G47230	AtERF5	38	53	180	3.00E-48		
EcAP2/ERF3	AT5G47230	AtERF5	35	51	146	5.00E-38		
EcAP2/ERF4	AT5G47230	AtERF5	35	49	147	4.00E-38		
EcAP2/ERF5	AT1G13040		47	58	176	3.00E-47	○	
EcAP2/ERF6	AT4G17500	AtERF1	45	62	132	7.00E-34		
EcAP2/ERF7	AT5G53290	CRF3	35	52	189	9.00E-51		
EcAP2/ERF8	AT5G47230	AtERF5	42	56	190	3.00E-51	○	
EcAP2/ERF9	AT5G25190	ESE3	51	62	154	9.00E-41		○
EcAP2/ERF10	AT4G17490	ERF6	37	54	162	8.00E-43		
EcAP2/ERF11	AT1G04370	ERF14	60	75	157	1.00E-41	○	
EcAP2/ERF12	AT3G23240	ERF1	55	70	220	2.00E-60		
EcAP2/ERF13	AT2G41710		55	66	407	E-116	○	○
EcAP2/ERF14	AT5G44210	ERF9	41	55	112	5.00E-28		
EcAP2/ERF15	AT4G36920	AP2	85	93	341	E-101		
EcAP2/ERF16	AT4G18450	AtERF#091	32	50	126	6.00E-32	○	
EcAP2/ERF17	AT3G23240	ERF1	55	68	218	9.00E-60		
EcAP2/ERF18	AT4G17500	AtERF1	50	67	155	8.00E-41		
EcAP2/ERF19	AT3G23240	ERF1	58	71	227	2.00E-62	○	

EcAP2/ERF20	AT5G13330	RAP2.6L	48	58	166	4.00E-44	○	○
EcAP2/ERF21	AT1G28160	AtERF#087	41	54	177	3.00E-47	○	
EcAP2/ERF22	AT1G51120		55	68	296	5.00E-83	○	
EcAP2/ERF23	AT3G16770	EBP/RAP2.3	46	59	205	1.00E-55	○	○
EcAP2/ERF24	AT1G53910	RAP2.12	40	50	151	3.00E-39		
EcAP2/ERF25	AT5G13910	LEP	48	61	118	8.00E-30		
EcAP2/ERF26	AT4G34410	RRTF1	51	62	171	2.00E-45	○	○
EcAP2/ERF27	AT4G27950	CRF4	39	56	134	2.00E-34		
EcAP2/ERF28	AT5G05410	DREB2A	37	50	120	4.00E-42		
EcAP2/ERF29	AT3G25890	CRF11	34	52	134	4.00E-34	○	
EcAP2/ERF30	AT5G44210	ERF9	32	43	67	3.00E-14		
EcAP2/ERF31	AT1G28160	AtERF#087	46	60	167	3.00E-44		
EcAP2/ERF32	AT2G28550	RAP2.7	44	55	178	1.00E-47		
EcAP2/ERF33	AT1G28360	ERF12	43	51	132	4.00E-34	○	○
EcAP2/ERF34	AT4G31060	AtERF#015	41	62	133	2.00E-34	○	
EcAP2/ERF35	AT1G78080	RAP2.4	41	52	246	7.00E-68	○	
EcAP2/ERF36	AT1G53910	RAP2.12	44	57	205	1.00E-55		
EcAP2/ERF37	AT2G20880	ERF53	39	48	196	8.00E-53	○	○
EcAP2/ERF38	AT1G53910	RAP2.12	44	56	283	3.00E-79	○	
EcAP2/ERF39	AT4G27950	CRF4	42	58	157	2.00E-41		
EcAP2/ERF40	AT3G54320	WRI1	44	60	141	2.00E-36		
EcAP2/ERF41	AT1G50640	ERF3	50	58	189	4.00E-51		
EcAP2/ERF42	AT1G50640	ERF3	54	64	230	2.00E-63	○	
EcAP2/ERF43	AT3G15210	ERF4/RAP2.5	40	50	134	1.00E-34		
EcAP2/ERF44	AT1G78080	RAP2.4	56	67	207	1.00E-56		

EcAP2/ERF45	AT4G27950	CRF4	38	54	210	4.00E-57	○	
EcAP2/ERF46	AT1G24590	ESR2/DRNL	39	47	139	1.00E-35	○	
EcAP2/ERF47	AT1G25560	TEM3	59	68	368	E-104		
EcAP2/ERF48	AT3G15210	ERF4/RAP2.5	43	55	142	1.00E-36		
EcAP2/ERF49	AT1G19210	AtERF#017	45	56	155	7.00E-41		
EcAP2/ERF50	AT3G60490	AtERF#035	48	64	204	2.00E-55	○	
EcAP2/ERF51	AT1G24590	ESR2/ERNL	39	50	139	2.00E-35		
EcAP2/ERF52	AT3G15210	ERF4/RAP2.5	40	55	134	1.00E-34		
EcAP2/ERF53	AT4G23750	CRF2	36	51	186	5.00E-50		
EcAP2/ERF54	AT1G25560	TEM3	58	70	399	E-114	○	
EcAP2/ERF55	AT5G18560	PUCHI	47	55	185	2.00E-49	○	○
EcAP2/ERF56	AT1G53910	RAP2.12	44	55	269	8.00E-75		
EcAP2/ERF57	AT1G50640	ERF3	55	63	217	2.00E-59		
EcAP2/ERF58								
EcAP2/ERF59	AT1G64380	AtERF#061	41	55	214	2.00E-58	○	○
EcAP2/ERF60								
EcAP2/ERF61	AT1G80580	AtERF#084	44	57	145	6.00E-38	○	○
EcAP2/ERF62	AT4G23750	CRF2	36	51	169	8.00E-45		
EcAP2/ERF63	AT4G13620	AtERF#062	37	51	183	7.00E-49	○	○
EcAP2/ERF64	AT1G75490	DREB2D	45	60	165	8.00E-44	○	○
EcAP2/ERF65	AT5G25190	ESE3	54	66	191	1.00E-51	○	
EcAP2/ERF66	AT4G36900	RAP2.10	50	60	155	6.00E-41		
EcAP2/ERF67	AT5G67190	DEAR2	56	65	168	5.00E-45	○	
EcAP2/ERF68	AT1G71450	AtERF#021	50	61	167	2.00E-44	○	
EcAP2/ERF69	AT5G13910	LEP	45	56	180	4.00E-48	○	

EcAP2/ERF70	AT1G33760	AtERF#022	46	64	149	5.00E-39		
EcAP2/ERF71	AT5G11590	TINY2	57	66	200	2.00E-54		
EcAP2/ERF72	AT2G28550	RAP2.7	49	58	316	6.00E-89		○
EcAP2/ERF73	AT5G11590	TINY2	57	68	216	4.00E-59		
EcAP2/ERF74	AT5G11590	TINY2	51	63	214	1.00E-58		
EcAP2/ERF75	AT5G11590	TINY2	63	71	223	4.00E-61	○	
EcAP2/ERF76	AT2G36450	HRD	52	66	182	5.00E-49		
EcAP2/ERF77	AT3G60490	AtERF#035	48	64	202	6.00E-55		
EcAP2/ERF78	AT3G60490	AtERF#035	46	60	166	5.00E-44		
EcAP2/ERF79	AT1G01250	AtERF#023	59	65	186	3.00E-50	○	○
EcAP2/ERF80	AT5G25190	ESE3	56	66	180	2.00E-48		
EcAP2/ERF81	AT5G67190	DEAR2	54	62	154	1.00E-40		
EcAP2/ERF82	AT2G28550	RAP2.7	50	59	381	E-108		
EcAP2/ERF83	AT5G51990	CBF4/DREB1D	55	66	222	7.00E-61	○	
EcAP2/ERF84	AT5G51990	CBF4/DREB1D	52	64	207	1.00E-56		
EcAP2/ERF85	AT5G51990	CBF4/DREB1D	54	65	184	1.00E-49		
EcAP2/ERF86	AT5G51990	CBF4/DREB1D	54	64	208	1.00E-56		
EcAP2/ERF87	AT1G63040	AtERF#026	54	67	193	4.00E-52	○	
EcAP2/ERF88	AT1G77640	AtERF#013	49	61	161	1.00E-42		
EcAP2/ERF89	AT1G44830	AtERF#014	47	56	183	3.00E-49		
EcAP2/ERF90	AT1G44830	AtERF#014	48	60	188	9.00E-51	○	
EcAP2/ERF91	AT2G36450	HRD	60	72	192	5.00E-52	○	○
EcAP2/ERF92	AT5G51990	CBF4/DREB1D	53	68	185	6.00E-50		
EcAP2/ERF93	AT1G63040	AtERF#026	50	64	190	2.00E-51		
EcAP2/ERF94	AT3G57600	DREB2F	53	68	272	7.00E-76	○	○

EcAP2/ERF95	AT1G68550	CRF10	33	46	122	8.00E-31		
EcAP2/ERF96	AT5G51990	CBF4/DREB1D	51	64	183	3.00E-49		
EcAP2/ERF97	AT5G51990	CBF4/DREB1D	53	65	203	3.00E-55		
EcAP2/ERF98	AT1G15360	WIN1/SHN1	58	72	221	9.00E-61	○	○
EcAP2/ERF99	AT5G51990	CBF4/DREB1D	52	61	189	5.00E-51		
EcAP2/ERF100	AT5G51990	CBF4/DREB1D	52	64	198	8.00E-54		
EcAP2/ERF101	AT2G40220	ABI4	40	55	225	1.00E-61	○	
EcAP2/ERF102	AT4G31060	AtERF#015	41	58	129	3.00E-33		
EcAP2/ERF103	AT1G19210	AtERF#017	49	61	169	6.00E-45	○	
EcAP2/ERF104	AT2G40340	DREB2C	35	49	165	1.00E-43		
EcAP2/ERF105	AT1G51190	PLT2	52	66	243	3.00E-67		
EcAP2/ERF106	AT5G17430	BBM	62	77	252	7.00E-70		
EcAP2/ERF107	AT5G65510	AIL7	44	57	220	4.00E-60		
EcAP2/ERF108	AT5G10510	AIL6	43	54	396	E-113	○	○
EcAP2/ERF109	AT4G37750	ANT	50	64	447	E-128	○	○
EcAP2/ERF110	AT2G28550	RAP2.7	50	61	385	E-110	○	
EcAP2/ERF111	AT4G37750	ANT	37	49	277	5.00E-77		
EcAP2/ERF112	AT5G57390	AIL5	47	56	444	E-127		○
EcAP2/ERF113	AT1G51190	PLT2	47	57	452	E-129		
EcAP2/ERF114	AT4G23750	CRF2	31	43	107	4.00E-26		
EcAP2/ERF115	AT3G20840	PLT1	41	56	151	3.00E-39		
EcAP2/ERF116	AT1G49120	CRF9	28	56	54	1.00E-10		
EcAP2/ERF117								
EcAP2/ERF118	AT1G03800	ERF10	33	44	57	2.00E-11		
EcAP2/ERF119	AT1G68840	RAV2	57	70	369	E-105		

EcAP2/ERF120	AT3G20310	ERF7	38	53	55	7.00E-11		
EcAP2/ERF121	AT1G25560	TEM3	60	69	394	E-112		
EcAP2/ERF122	AT3G54320	WRI1	51	61	324	2.00E-91	○	○
EcAP2/ERF123	AT1G16060	ADAP	44	53	250	3.00E-69		
EcAP2/ERF124	AT4G36920	AP2	45	54	400	E-114	○	
EcAP2/ERF125	AT5G19790	RAP2.11	43	53	197	3.00E-53	○	○
EcAP2/ERF126	AT4G23750	CRF2	38	53	202	1.00E-54		
EcAP2/ERF127								
EcAP2/ERF128	AT1G03800	ERF10	34	47	67	2.00E-14		
EcAP2/ERF129	AT2G40340	DREB2C	36	50	181	2.00E-48		
EcAP2/ERF130	AT2G40340	DREB2C	38	51	199	1.00E-53	○	
EcAP2/ERF131	AT2G40220	ABI4	40	53	177	2.00E-47		
EcAP2/ERF132	AT1G19210	AtERF#017	47	58	148	8.00E-39		
EcAP2/ERF133	AT1G03800	ERF10	38	49	162	2.00E-14		
EcAP2/ERF134	AT3G20310	ERF7	43	56	59	5.00E-12		

Supplementary Table S2

A comparison of genes percent in each family of *EcAP2/ERF* with other plant species.

Plant specie		AP2	RAV	Solo	DREB	ERF	Total	Genome size (Mb)	Gene density (per Mb)	Reference
Eschscholzia californica	Gene number	20	5	2	47	60	134	502	0.267	This study
	Percent	14.93	3.73	1.49	35.07	44.78				
Arabidopsis thaliana	Gene number	18	6	1	57	65	147	125	1.176	Nakano et al., 2006[16]
	Percent	12.24	4.08	0.68	38.78	44.22				
Vitis vinifera	Gene number	20	6	1	40	82	149	490	0.304	Licausi et al., 2010[32]
	Percent	13.42	4.03	0.67	26.85	55.03				
Medicago truncatula	Gene number	21	3	1	50	48	123	465	0.265	Shu et al., 2016[59]
	Percent	17.07	2.44	0.81	40.65	39.02				
Ricinus communis	Gene number	19	4	1	34	56	114	310	0.368	Xu et al., 2013[25]
	Percent	16.67	3.51	0.88	29.82	49.12				
Ziziphus jujuba	Gene number	17	5	1	39	57	119	360	0.331	Zhang and Li, 2018[35]
	Percent	14.29	4.20	0.84	32.77	47.90				
Populus trichocarpa	Gene number	26	5	1	77	91	200	485	0.412	Zhuang et al., 2008[37]
	Percent	13.00	2.50	0.50	38.50	45.50				
Brassica rapa ssp. *pekinensis*	Gene number	30	14	1	105	131	281	485	0.579	Liu et al., 2013[38]
	Percent	10.68	4.98	0.36	37.37	46.62				
Daucus carota	Gene number	38	12	3	69	145	267	480	0.556	Li et al., 2015[34]
	Percent	14.23	4.49	1.12	25.84	54.31				

Oryza sativa L. *japonica*	Gene number	24	5	0	57	77	163	389	0.419	Sharoni et al., 2011[33]
	Percent	14.72	3.07	0.00	34.97	47.24				
Brachypodium distachyon	Gene number	24	4	1	52	60	141	355	0.397	Cui et al., 2016[27]
	Percent	17.02	2.84	0.71	36.88	42.55				
Setaria italica	Gene number	28	5	0	48	90	171	515	0.332	Lata et al., 2014[60]
	Percent	16.37	2.92	0.00	28.07	52.63				
Phyllostachys edulis	Gene number	28	7	1	27	53	116	2075	0.056	Wu et al., 2015[26]
	Percent	24.14	6.03	0.86	23.28	45.69				

59. Shu, Y., Liu, Y., Zhang, J., Song, L. & Guo, C. Genome-Wide Analysis of the AP2/ERF Superfamily Genes and their Responses to Abiotic Stress in *Medicago truncatula*. *Front. Plant. Sci.* **6**, 1247, https://doi.org/10.3389/fpls.2015.01247 (2015).

60. Lata, C. *et al*. Genome-wide investigation and expression profiling of AP2/ERF transcription factor superfamily in foxtail millet (*Setaria italica* L.). *PLoS One* **9**, e113092, https://doi.org/10.1371/journal.pone.0113092 (2014).

Supplementary Table S3

A location of *EcAP2/ERF* genes in each scaffold.

Scaffold	Genes							
Eca_sc000102.1	*EcAP2/ERF45*							
Eca_sc000108.1	*EcAP2/ERF97*	*EcAP2/ERF99*	*EcAP2/ERF100*					
Eca_sc000145.1	*EcAP2/ERF25*							
Eca_sc000153.1	*EcAP2/ERF108*							
Eca_sc000212.1	*EcAP2/ERF117*							
Eca_sc000360.1	*EcAP2/ERF7*	*EcAP2/ERF6*						
Eca_sc000388.1	*EcAP2/ERF83*	*EcAP2/ERF84*	*EcAP2/ERF85*	*EcAP2/ERF86*				
Eca_sc000399.1	*EcAP2/ERF67*							
Eca_sc000448.1	*EcAP2/ERF1*							
Eca_sc000462.1	*EcAP2/ERF65*							
Eca_sc000537.1	*EcAP2/ERF129*	*EcAP2/ERF134*	*EcAP2/ERF60*	*EcAP2/ERF120*	*EcAP2/ERF118*	*EcAP2/ERF116*	*EcAP2/ERF128*	*EcAP2/ERF133*
Eca_sc000587.1	*EcAP2/ERF78*	*EcAP2/ERF124*						
Eca_sc000630.1	*EcAP2/ERF75*	*EcAP2/ERF76*						
Eca_sc000631.1	*EcAP2/ERF37*							
Eca_sc000635.1	*EcAP2/ERF119*							
Eca_sc000661.1	*EcAP2/ERF39*							
Eca_sc001060.1	*EcAP2/ERF94*							
Eca_sc001096.1	*EcAP2/ERF96*							
Eca_sc001139.1	*EcAP2/ERF54*	*EcAP2/ERF53*						

Eca_sc001224.1	*EcAP2/ERF91*	*EcAP2/ERF92*	
Eca_sc001253.1	*EcAP2/ERF10*		
Eca_sc001301.1	*EcAP2/ERF74*		
Eca_sc001479.1	*EcAP2/ERF114*		
Eca_sc001493.1	*EcAP2/ERF125*		
Eca_sc001536.1	*EcAP2/ERF87*		
Eca_sc001539.1	*EcAP2/ERF110*		
Eca_sc001713.1	*EcAP2/ERF73*		
Eca_sc001754.1	*EcAP2/ERF81*		
Eca_sc001803.1	*EcAP2/ERF104*		
Eca_sc001904.1	*EcAP2/ERF57*		
Eca_sc001921.1	*EcAP2/ERF44*		
Eca_sc001928.1	*EcAP2/ERF27*	*EcAP2/ERF28*	*EcAP2/ERF26*
Eca_sc001936.1	*EcAP2/ERF52*		
Eca_sc002196.1	*EcAP2/ERF90*		
Eca_sc002218.1	*EcAP2/ERF43*		
Eca_sc002317.1	*EcAP2/ERF122*		
Eca_sc002391.1	*EcAP2/ERF98*		
Eca_sc002447.1	*EcAP2/ERF95*		
Eca_sc002690.1	*EcAP2/ERF113*		
Eca_sc002983.1	*EcAP2/ERF106*	*EcAP2/ERF107*	
Eca_sc003030.1	*EcAP2/ERF19*		
Eca_sc003413.1	*EcAP2/ERF56*		

Eca_sc003743.1	*EcAP2/ERF80*		
Eca_sc003758.1	*EcAP2/ERF103*	*EcAP2/ERF123*	
Eca_sc003765.1	*EcAP2/ERF131*	*EcAP2/ERF130*	
Eca_sc003774.1	*EcAP2/ERF42*	*EcAP2/ERF41*	
Eca_sc003794.1	*EcAP2/ERF8*		
Eca_sc003893.1	*EcAP2/ERF59*		
Eca_sc003926.1	*EcAP2/ERF77*		
Eca_sc004205.1	*EcAP2/ERF109*		
Eca_sc004328.1	*EcAP2/ERF101*		
Eca_sc004486.1	*EcAP2/ERF72*	*EcAP2/ERF18*	
Eca_sc005351.1	*EcAP2/ERF112*		
Eca_sc005409.1	*EcAP2/ERF111*		
Eca_sc006292.1	*EcAP2/ERF3*	*EcAP2/ERF4*	*EcAP2/ERF2*
Eca_sc006305.1	*EcAP2/ERF36*		
Eca_sc007884.1	*EcAP2/ERF35*		
Eca_sc010606.1	*EcAP2/ERF51*		
Eca_sc010932.1	*EcAP2/ERF40*		
Eca_sc011255.1	*EcAP2/ERF34*		
Eca_sc013037.1	*EcAP2/ERF62*		
Eca_sc014577.1	*EcAP2/ERF30*		
Eca_sc183659.1	*EcAP2/ERF16*		
Eca_sc184725.1	*EcAP2/ERF33*		
Eca_sc184896.1	*EcAP2/ERF121*		

Eca_sc186325.1	*EcAP2/ERF15*		
Eca_sc186764.1	*EcAP2/ERF132*		
Eca_sc186929.1	*EcAP2/ERF89*		
Eca_sc194094.1	*EcAP2/ERF23*	*EcAP2/ERF24*	
Eca_sc194316.1	*EcAP2/ERF17*		
Eca_sc194480.1	*EcAP2/ERF126*		
Eca_sc194483.1	*EcAP2/ERF58*		
Eca_sc194486.1	*EcAP2/ERF21*	*EcAP2/ERF20*	*EcAP2/ERF22*
Eca_sc194541.1	*EcAP2/ERF69*	*EcAP2/ERF68*	
Eca_sc194561.1	*EcAP2/ERF61*		
Eca_sc194565.1	*EcAP2/ERF71*		
Eca_sc194570.1	*EcAP2/ERF93*		
Eca_sc194602.1	*EcAP2/ERF79*		
Eca_sc194610.1	*EcAP2/ERF64*		
Eca_sc194615.1	*EcAP2/ERF25*		
Eca_sc194620.1	*EcAP2/ERF70*		
Eca_sc194641.1	*EcAP2/ERF14*	*EcAP2/ERF11*	*EcAP2/ERF12*
Eca_sc194665.1	*EcAP2/ERF102*		
Eca_sc194668.1	*EcAP2/ERF48*	*EcAP2/ERF50*	*EcAP2/ERF49*
Eca_sc194672.1	*EcAP2/ERF115*		
Eca_sc194674.1	*EcAP2/ERF9*		
Eca_sc194677.1	*EcAP2/ERF66*		
Eca_sc194689.1	*EcAP2/ERF5*		

Eca_sc194692.1	*EcAP2/ERF29*	
Eca_sc194696.1	*EcAP2/ERF47*	*EcAP2/ERF46*
Eca_sc194697.1	*EcAP2/ERF13*	
Eca_sc194713.1	*EcAP2/ERF63*	
Eca_sc194718.1	*EcAP2/ERF55*	
Eca_sc194732.1	*EcAP2/ERF31*	
Eca_sc194755.1	*EcAP2/ERF38*	
Eca_sc194756.1	*EcAP2/ERF88*	
Eca_sc194794.1	*EcAP2/ERF32*	

Supplementary Table S4

Validation of *AP2/ERF* transcription factors of *E. californica* by BLAST.

Name	Protein length	BLASTn, Eschscholzia californica (using Phytometasyn database)	E-value	Predicted protein length	BLASTx, Arabidopsis thaliana	E-value	Protein length	BLASTx, other BIA-producing plants	E-value	Protein length
**EcAP2/ERF1**	222	MAGPIE:eca_ECARTPF1_assembled--ECARTPF1_rep_c19853	0	222	ethylene responsive element binding factor 1	2E-35	266	ethylene-responsive transcription factor 1-like [Papaver somniferum]	2E-28	273
**EcAP2/ERF2**	322	MAGPIE:eca_ECARTPF1_assembled--ECARTPF1_rep_c166	0	322	ethylene responsive element binding factor 5	4E-27	300	AP2/ERF domain [Macleaya cordata]	8E-51	345
**EcAP2/ERF3**	303	MAGPIE:eca_ECARTPF1_assembled--ECARTPF1_rep_c512	0	305	ethylene responsive element binding factor 5	2E-29	300	AP2/ERF domain [Macleaya cordata]	2E-55	356
**EcAP2/ERF4**	301	MAGPIE:eca_ECARTPF1_assembled--ECARTPF1_rep_c196	0	303	ethylene responsive element binding factor 5	4E-25	300	AP2/ERF domain [Macleaya cordata]	5E-51	345
**EcAP2/ERF5**	230	MAGPIE:eca_ECARTPF1_Velvet--Contig12874	0	162	hypothetical protein AXX17_AT4G14710	1E-40	223	PREDICTED: ethylene-responsive transcription factor-like protein At4g13040 isoform X2 [Nelumbo nucifera]	7E-82	232
**EcAP2/ERF6**	188	MAGPIE:eca_ECARTPF1_Velvet--Contig19883	0	185	ethylene responsive element binding factor 2	8E-27	243	AP2/ERF domain [Macleaya cordata]	4E-47	186
**EcAP2/ERF7**	376	MAGPIE:eca_ECARTPF1_Velvet--Contig12079	7E-25	351	cytokinin response factor 4	1E-42	335	PREDICTED: ethylene-responsive transcription factor CRF3 [Nelumbo nucifera]	5E-58	328
**EcAP2/ERF8**	312	MAGPIE:eca_ECARTPF1_Velvet--Contig26026	0	312	ethylene responsive element binding factor 5	3E-36	300	AP2/ERF domain [Macleaya cordata]	3E-81	345
**EcAP2/ERF9**	145	MAGPIE:eca_ECARTPF1_Velvet--Contig3216	2E-33	445	SHN2	1E-22	187	AP2/ERF domain [Macleaya cordata]	3E-56	148
**EcAP2/ERF10**	271	MAGPIE:eca_ECARTPF1_Velvet--Contig19629	0	271	ethylene responsive element binding factor 6	9E-37	282	AP2/ERF domain [Macleaya cordata]	6E-76	345
**EcAP2/ERF11**	133	MAGPIE:eca_ECARTPF1_Velvet--Singlet5881	0	133	TDR1	8E-23	139	AP2/ERF domain [Macleaya cordata]	6E-27	136
**EcAP2/ERF12**	214	MAGPIE:eca_ECARTPF1_Velvet--Contig27317	0	214	ethylene response factor 1	9E-50	218	PREDICTED: ethylene-responsive transcription factor 1B-like [Nelumbo nucifera]	1E-68	230
**EcAP2/ERF13**	439	MAGPIE:eca_ECARTPF1_Velvet--Contig25305	0	415	putative AP2 domain transcription factor	7E-103	423	AP2-like ethylene-responsive transcription factor At2g41710 [Papaver somniferum]	4E-158	440

**EcAP2/ERF14**	199	MAGPIE:eca_ECARTPF1_assembled--ECARTPF1_rep_c10870	0	201	ethylene response factor 7	3E-24	244	AP2/ERF domain [Macleaya cordata]	1E-47	229
**EcAP2/ERF15**	367	MAGPIE:eca_ECARTPF1_Velvet--Contig5418	1E-173	389	FLO2	7E-104	432	AP2/ERF domain [Macleaya cordata]	3E-131	496
**EcAP2/ERF16**	275	MAGPIE:eca_ECARTPF1_Velvet--Singlet43578	0	275	ethylene response factor ERF1	9E-22	218	AP2/ERF domain [Macleaya cordata]	2E-116	275
**EcAP2/ERF17**	221	MAGPIE:eca_ECARTPF1_Velvet--Contig23624	0	221	ethylene response factor 1	8E-55	218	AP2/ERF domain [Macleaya cordata]	4E-88	217
**EcAP2/ERF18**	296	MAGPIE:eca_ECARTPF1_assembled--ECARTPF1_rep_c1664	0	295	ethylene responsive element binding factor 1	2E-30	268	AP2/ERF domain [Macleaya cordata]	5E-72	324
**EcAP2/ERF19**	211	MAGPIE:eca_ECARTPF1_Velvet--Contig5077	0	211	ethylene response factor 1	2E-70	218	AP2/ERF domain [Macleaya cordata]	7E-80	216
**EcAP2/ERF20**	267	MAGPIE:eca_ECARTPF1_Velvet--Contig28396	0	266	Integrase-type DNA-binding superfamily protein	4E-33	263	PREDICTED: ethylene-responsive transcription factor ERF113 [Nelumbo nucifera]	4E-46	329
**EcAP2/ERF21**	306	MAGPIE:eca_ECARTPF1_Velvet--Singlet43578	9E-23	275	putative AP2/EREBP transcription factor	1E-27	245	AP2/ERF domain [Macleaya cordata]	9E-66	318
**EcAP2/ERF22**	365	MAGPIE:eca_ECARTPF1_Velvet--Contig3480	0	349	AP2/B3 transcription factor family protein	5E-97	352	AP2/ERF domain [Macleaya cordata]	1E-149	389
**EcAP2/ERF23**	274	MAGPIE:eca_ECARTPF1_Velvet--Contig8796	0	274	RAP2.3	3E-43	246	AP2/ERF domain [Macleaya cordata]	2E-86	264
**EcAP2/ERF24**	442	MAGPIE:eca_ECARTPF1_assembled--ECARTPF1_rep_c792	0	327	AP2 domain containing protein, putative	2E-26	358	uncharacterized protein LOC113316800 isoform X3 [Papaver somniferum]	5E-65	410
**EcAP2/ERF25**	154	MAGPIE:eca_ECARTPF1_Velvet--Contig25136	4E-11	227	Integrase-type DNA-binding superfamily protein	3E-30	219	AP2/ERF domain [Macleaya cordata]	3E-38	150
**EcAP2/ERF26**	286	MAGPIE:eca_ECARTPF1_assembled--ECARTPF1_rep_c498	0	286	RRTF1	2E-25	268	ethylene-responsive transcription factor ERF109-like [Papaver somniferum]	3E-44	289
**EcAP2/ERF27**	246	MAGPIE:eca_ECARTPF1_Velvet--Contig15974	0	246	CRF4	3E-20	335	AP2/ERF domain [Macleaya cordata]	5E-67	254
**EcAP2/ERF28**	317	MAGPIE:eca_ECARTPF1_Velvet--Contig28265	1E-59	355	Integrase-type DNA-binding superfamily protein	1E-34	298	hypothetical protein AQUCO_03000214v1 [Aquilegia coerulea]	6E-43	323
**EcAP2/ERF29**	338	MAGPIE:eca_ECARTPF1_Velvet--Singlet30412	0	337	Integrase-type DNA-binding superfamily protein	3E-16	262	ethylene-responsive transcription factor ERF118-like [Papaver somniferum]	4E-48	388

**EcAP2/ERF30**	184	MAGPIE:eca_ECARTPF1_Velvet--Contig23909	1E-12	186	ERF domain protein 10	7E-14	245	PREDICTED: ethylene-responsive transcription factor 9-like [Nelumbo nucifera]	1E-11	175
**EcAP2/ERF31**	295	MAGPIE:eca_ECARTPF1_Velvet--Singlet39940	7E-24	169	putative AP2/EREBP transcription factor	7E-32	245	AP2/ERF domain [Macleaya cordata]	1E-74	318
**EcAP2/ERF32**	362	MAGPIE:eca_ECARTPF1_Velvet--Contig23931	0	265	related to AP2.7	2E-47	283	AP2/ERF domain [Macleaya cordata]	6E-88	517
**EcAP2/ERF33**	169	MAGPIE:eca_ECARTPF1_Velvet--Singlet39940	0	169	ERF domain protein 12	6E-35	189	PREDICTED: ethylene-responsive transcription factor 12-like [Nelumbo nucifera]	7E-45	157
**EcAP2/ERF34**	181	MAGPIE:eca_ECARTPF1_Velvet--Singlet44397	1E-12	112	Integrase-type DNA-binding superfamily protein	2E-33	187	AP2/ERF domain [Macleaya cordata]	1E-72	184
**EcAP2/ERF35**	433	MAGPIE:eca_ECARTPF1_assembled--ECARTPF1_rep_c1798	0	416	RAP2.4	4E-41	261	AP2/ERF domain [Macleaya cordata]	4E-90	379
**EcAP2/ERF36**	353	MAGPIE:eca_ECARTPF1_assembled--ECARTPF1_rep_c609	0	375	AP2 domain containing protein, putative	6E-47	358	AP2/ERF domain [Macleaya cordata]	1E-134	421
**EcAP2/ERF37**	406	MAGPIE:eca_ECARTPF1_Velvet--Contig10577	0	408	ERF53	6E-45	336	AP2/ERF domain [Macleaya cordata]	1E-134	405
**EcAP2/ERF38**	384	MAGPIE:eca_ECARTPF1_Velvet--Contig27210	0	384	related to AP2 2	2E-61	374	ethylene-responsive transcription factor RAP2-12-like isoform X2 [Papaver somniferum]	1E-142	396
**EcAP2/ERF39**	261	MAGPIE:eca_ECARTPF1_Velvet--Contig28418	0	279	putative AP2/EREBP transcription factor	1E-19	334	AP2/ERF domain [Macleaya cordata]	2E-92	254
**EcAP2/ERF40**	325	MAGPIE:eca_ECARTPF1_assembled--ECARTPF1_c15764	0.00008	211	WRI1	1E-29	365	hypothetical protein AQUCO_07400065v1 [Aquilegia coerulea]	3E-27	337
**EcAP2/ERF41**	210	MAGPIE:eca_ECARTPF1_assembled--ECARTPF1_rep_c6774	0	211	AP2 domain containing protein RAP2.5	1E-18	213	AP2/ERF domain [Macleaya cordata]	2E-20	225
**EcAP2/ERF42**	231	MAGPIE:eca_ECARTPF1_Velvet--Contig28549	0	233	ERF3	3E-32	225	PREDICTED: ethylene-responsive transcription factor 3-like [Nelumbo nucifera]	1E-36	235
**EcAP2/ERF43**	235	MAGPIE:eca_ECARTPF1_assembled--ECARTPF1_rep_c4506	0	237	ERF9	2E-22	200	ethylene-responsive transcription factor 4-like [Papaver somniferum]	1E-26	271
**EcAP2/ERF44**	376	MAGPIE:eca_ECARTPF1_Velvet--Singlet31422	0	376	WIND1	6E-39	333	AP2/ERF domain [Macleaya cordata]	7E-58	409
**EcAP2/ERF45**	349	MAGPIE:eca_ECARTPF1_Velvet--Contig12079	0	351	cytokinin response factor 2	4E-36	343	AP2/ERF domain [Macleaya cordata]	3E-75	344

**EcAP2/ERF46**	379	MAGPIE:eca_ECARTPF1_Velvet--Contig26952	2E-13	256	DORNROSCHEN-like protein	1E-11	306	ethylene-responsive transcription factor ESR1-like	1E-24	449
**EcAP2/ERF47**	368	MAGPIE:eca_ECARTPF1_Velvet--Singlet42715	0	368	AP2/B3 transcription factor family protein	8E-127	361	AP2/ERF domain [Macleaya cordata]	3E-180	359
**EcAP2/ERF48**	247	MAGPIE:eca_ECARTPF1_assembled--ECARTPF1_rep_c1273	0	248	ERF9	1E-22	200	AP2/ERF domain [Macleaya cordata]	2E-46	242
**EcAP2/ERF49**	218	MAGPIE:eca_ECARTPF1_Velvet--Contig12500	0	220	Integrase-type DNA-binding superfamily protein	6E-34	185	AP2/ERF domain [Macleaya cordata]	4E-46	229
**EcAP2/ERF50**	235	MAGPIE:eca_ECARTPF1_Velvet--Contig26015	0	238	putative AP2 domain transcription factor	4E-46	295	ethylene-responsive transcription factor ERF039-like [Papaver somniferum]	9E-61	252
**EcAP2/ERF51**	426	MAGPIE:eca_ECARTPF1_Velvet--Contig26952	9E-12	256	DORNROSCHEN-like protein	1E-11	306	AP2/ERF domain [Macleaya cordata]	7E-20	469
**EcAP2/ERF52**	224	MAGPIE:eca_ECARTPF1_assembled--ECARTPF1_rep_c2226	0	234	ERF9	6E-23	200	AP2/ERF domain [Macleaya cordata]	3E-45	242
**EcAP2/ERF53**	353	MAGPIE:eca_ECARTPF1_Velvet--Contig12079	3E-29	351	cytokinin response factor 4	3E-44	335	PREDICTED: ethylene-responsive transcription factor CRF1-like [Nelumbo nucifera]	2E-56	339
**EcAP2/ERF54**	370	MAGPIE:eca_ECARTPF1_Velvet--Contig10421	0	370	AP2/B3 transcription factor family protein	9E-137	361	AP2/ERF domain [Macleaya cordata]	1E-167	387
**EcAP2/ERF55**	377	MAGPIE:eca_ECARTPF1_Velvet--Contig5077	7E-19	211	Integrase-type DNA-binding superfamily protein	2E-44	348	ethylene-responsive transcription factor ERF086-like [Papaver somniferum]	9E-109	368
**EcAP2/ERF56**	361	MAGPIE:eca_ECARTPF1_Velvet--Contig8599	0	367	AP2 domain containing protein, putative	3E-69	358	ethylene-responsive transcription factor RAP2-12-like isoform X2 [Papaver somniferum]	1E-147	396
**EcAP2/ERF57**	221	MAGPIE:eca_ECARTPF1_assembled--ECARTPF1_rep_c2401	0	206	ethylene responsive element binding factor 3	8E-22	225	AP2/ERF domain [Macleaya cordata]	1E-28	225
**EcAP2/ERF58**	193	MAGPIE:eca_ECARTPF1_Velvet--Contig23909	2E-28	186	AP2 domain containing protein RAP2.5	8E-27	213	ethylene-responsive transcription factor 12-like [Papaver somniferum]	1E-23	232
**EcAP2/ERF59**	281	MAGPIE:eca_ECARTPF1_Velvet--Singlet31787	0	280	Integrase-type DNA-binding superfamily protein	6E-32	335	AP2/ERF domain [Macleaya cordata]	3E-76	291
**EcAP2/ERF60**	143	MAGPIE:eca_ECARTPF1_Velvet--Singlet12614	0.06	228	Integrase-type DNA-binding superfamily protein	7E-10	219	AP2/ERF domain [Macleaya cordata]	2E-07	150
**EcAP2/ERF61**	218	MAGPIE:eca_ECARTPF1_Velvet--Singlet8824	2E-17	254	hypothetical protein AXX17_AT1G75430	5E-21	255	AP2/ERF domain [Macleaya cordata]	4E-31	295

**EcAP2/ERF62**	346	MAGPIE:eca_ECARTPF1_Velvet--Contig12079	9E-30	351	cytokinin response factor 4	5E-39	335	PREDICTED: ethylene-responsive transcription factor CRF1-like [Nelumbo nucifera]	9E-53	339
**EcAP2/ERF63**	415	MAGPIE:eca_ECARTPF1_Velvet--Singlet31787	3E-36	281	Integrase-type DNA-binding superfamily protein	1E-34	388	ethylene-responsive transcription factor ERF062-like [Papaver somniferum]	8E-57	432
**EcAP2/ERF64**	231	MAGPIE:eca_ECARTPF1_Velvet--Singlet37091	5E-37	281	Integrase-type DNA-binding superfamily protein	1E-39	206	PREDICTED: dehydration-responsive element-binding protein 2C-like [Nelumbo nucifera]	1E-38	204
**EcAP2/ERF65**	194	MAGPIE:eca_ECARTPF1_assembled--ECARTPF1_rep_c3880	1E-17	155	Integrase-type DNA-binding superfamily protein	1E-40	181	PREDICTED: ethylene-responsive transcription factor ERF003-like [Nelumbo nucifera]	3E-61	187
**EcAP2/ERF66**	192	MAGPIE:eca_ECARTPF1_Velvet--Contig12709	0	152	DREB and EAR motif protein 2	1E-43	184	AP2/ERF domain [Macleaya cordata]	2E-56	196
**EcAP2/ERF67**	150	MAGPIE:eca_ECARTPF1_Velvet--Contig18746	0	175	DEAR2	2E-49	184	AP2/ERF domain [Macleaya cordata]	4E-65	142
**EcAP2/ERF68**	193	MAGPIE:eca_ECARTPF1_Velvet--Contig4816	0	205	Integrase-type DNA-binding superfamily protein	6E-45	183	ethylene-responsive transcription factor ERF021-like [Papaver somniferum]	2E-65	194
**EcAP2/ERF69**	272	MAGPIE:eca_ECARTPF1_Velvet--Singlet43578	2E-18	275	Integrase-type DNA-binding superfamily protein	3E-34	211	PREDICTED: ethylene-responsive transcription factor LEP-like [Nelumbo nucifera]	5E-48	275
**EcAP2/ERF70**	180	MAGPIE:eca_ECARTPF1_Velvet--Contig4816	3E-39	205	Integrase-type DNA-binding superfamily protein	4E-45	183	AP2/ERF domain [Macleaya cordata]	2E-72	186
**EcAP2/ERF71**	233	MAGPIE:eca_ECARTPF1_Velvet--Contig11049	2E-100	235	TINY2	5E-54	236	PREDICTED: dehydration-responsive element-binding protein 3-like [Nelumbo nucifera]	4E-76	237
**EcAP2/ERF72**	477	MAGPIE:eca_ECARTPF1_assembled--ECARTPF1_rep_c1195	0	478	related to AP2.7	1E-89	464	floral homeotic protein APETALA 2-like isoform X1 [Papaver somniferum]	8E-124	516
**EcAP2/ERF73**	240	MAGPIE:eca_ECARTPF1_Velvet--Contig11050	0	235	Integrase-type DNA-binding superfamily protein	4E-58	236	ethylene-responsive transcription factor ERF043-like [Papaver somniferum]	4E-74	253
**EcAP2/ERF74**	292	MAGPIE:eca_ECARTPF1_Velvet--Singlet32235	0	284	TINY2	6E-62	236	AP2/ERF domain [Macleaya cordata]	5E-88	246
**EcAP2/ERF75**	267	MAGPIE:eca_ECARTPF1_Velvet--Contig11048	0	267	Integrase-type DNA-binding superfamily protein	5E-64	236	PREDICTED: dehydration-responsive element-binding protein 3-like [Nelumbo nucifera]	3E-73	237
**EcAP2/ERF76**	201	MAGPIE:eca_ECARTPF1_Velvet--Contig22485	2E-21	226	Integrase-type DNA-binding superfamily protein	1E-43	184	AP2/ERF domain [Macleaya cordata]	4E-63	203
**EcAP2/ERF77**	238	MAGPIE:eca_ECARTPF1_Velvet--Contig26015	0	238	hypothetical protein AXX17_AT2G42530	3E-47	295	AP2/ERF domain [Macleaya cordata]	7E-69	255

**EcAP2/ERF78**	251	MAGPIE:eca_ECARTPF1_Velvet--Contig11048	1E-44	267	Integrase-type DNA-binding superfamily protein	1E-39	218	ethylene-responsive transcription factor ERF039-like [Papaver somniferum]	6E-48	252
**EcAP2/ERF79**	184	MAGPIE:eca_ECARTPF1_assembled--ECARTPF1_c5787	0	184	Similar to transcription factor TINY	1E-53	187	AP2/ERF domain [Macleaya cordata]	7E-68	179
**EcAP2/ERF80**	173	MAGPIE:eca_ECARTPF1_assembled--ECARTPF1_rep_c1061	2E-14	383	Integrase-type DNA-binding superfamily protein	2E-36	181	PREDICTED: ethylene-responsive transcription factor ERF003-like [Nelumbo nucifera]	8E-45	187
**EcAP2/ERF81**	153	MAGPIE:eca_ECARTPF1_Velvet--Contig9805	0	153	DEAR2	4E-48	184	AP2/ERF domain [Macleaya cordata]	1E-80	142
**EcAP2/ERF82**	511	MAGPIE:eca_ECARTPF1_Velvet--Contig25908	0	512	related to AP2.7	1E-106	464	floral homeotic protein APETALA 2-like isoform X1 [Papaver somniferum]	9E-163	536
**EcAP2/ERF83**	233	MAGPIE:eca_ECARTPF1_assembled--ECARTPF1_rep_c3664	0	233	CRT/DRE binding factor 2	6E-71	216	AP2/ERF domain [Macleaya cordata]	3E-97	228
**EcAP2/ERF84**	245	MAGPIE:eca_ECARTPF1_Velvet--Contig11460	0	238	C-repeat-binding factor 4	4E-63	224	dehydration-responsive element-binding protein 1A-like [Papaver somniferum]	2E-80	228
**EcAP2/ERF85**	205	MAGPIE:eca_ECARTPF1_Velvet--Contig11458	0	230	C-repeat-binding factor 4	6E-59	224	AP2/ERF domain [Macleaya cordata]	2E-86	228
**EcAP2/ERF86**	233	MAGPIE:eca_ECARTPF1_Velvet--Contig11460	0	238	C-repeat-binding factor 4	2E-67	224	dehydration-responsive element-binding protein 1A-like [Papaver somniferum]	1E-79	228
**EcAP2/ERF87**	254	MAGPIE:eca_ECARTPF1_assembled--ECARTPF1_rep_c42	0	255	RecName: Full=Ethylene-responsive transcription factor ERF026	4E-23	248	AP2/ERF domain [Macleaya cordata]	2E-41	242
**EcAP2/ERF88**	275	MAGPIE:eca_ECARTPF1_Velvet--Contig13517	0	280	Integrase-type DNA-binding superfamily protein	1E-28	244	ethylene-responsive transcription factor ERF014-like [Papaver somniferum]	1E-29	297
**EcAP2/ERF89**	280	MAGPIE:eca_ECARTPF1_Velvet--Contig14796	0	271	Integrase-type DNA-binding superfamily protein	2E-28	244	ethylene-responsive transcription factor ERF014-like [Papaver somniferum]	2E-29	298
**EcAP2/ERF90**	250	MAGPIE:eca_ECARTPF1_Velvet--Contig26777	0	250	Integrase-type DNA-binding superfamily protein	1E-27	230	PREDICTED: ethylene-responsive transcription factor ERF014-like [Nelumbo nucifera]	6E-30	221
**EcAP2/ERF91**	204	MAGPIE:eca_ECARTPF1_Velvet--Contig26235	2E-28	257	HRD	2E-36	184	AP2/ERF domain [Macleaya cordata]	5E-50	183
**EcAP2/ERF92**	217	MAGPIE:eca_ECARTPF1_Velvet--Contig22213	2E-93	172	C-repeat-binding factor 4	1E-41	224	dehydration-responsive element-binding protein 1F-like [Papaver somniferum]	2E-59	228
**EcAP2/ERF93**	203	MAGPIE:eca_ECARTPF1_assembled--ECARTPF1_rep_c9901	0	203	RecName: Full=Ethylene-responsive transcription factor ERF026	7E-24	248	AP2/ERF domain [Macleaya cordata]	2E-34	242

**EcAP2/ERF94**	278	MAGPIE:eca_ECARTPF1_Velvet--Singlet37091	3E-40	326	Integrase-type DNA-binding superfamily protein	2E-73	277	AP2/ERF domain [Macleaya cordata]	2E-129	285
**EcAP2/ERF95**	326	MAGPIE:eca_ECARTPF1_Velvet--Singlet42615	0	327	CRF1	4E-17	289	AP2/ERF domain [Macleaya cordata]	9E-49	349
**EcAP2/ERF96**	219	MAGPIE:eca_ECARTPF1_assembled--ECARTPF1_rep_c98	0	219	C-repeat binding factor 3	1E-51	216	dehydration-responsive element-binding protein 1C-like [Papaver somniferum]	3E-67	243
**EcAP2/ERF97**	213	MAGPIE:eca_ECARTPF1_Velvet--Singlet31552	0	233	C-repeat binding factor 2	3E-57	216	AP2/ERF domain [Macleaya cordata]	2E-81	228
**EcAP2/ERF98**	193	MAGPIE:eca_ECARTPF1_Velvet--Contig11850	3E-13	254	Integrase-type DNA-binding superfamily protein	2E-66	199	AP2/ERF domain [Macleaya cordata]	2E-89	200
**EcAP2/ERF99**	205	MAGPIE:eca_ECARTPF1_Velvet--Contig21023	0	237	C-repeat-binding factor 4	4E-61	224	AP2/ERF domain [Macleaya cordata]	4E-83	228
**EcAP2/ERF100**	232	MAGPIE:eca_ECARTPF1_Velvet--Singlet31553	0	233	C-repeat-binding factor 4	2E-60	224	AP2/ERF domain [Macleaya cordata]	1E-86	228
**EcAP2/ERF101**	373	MAGPIE:eca_ECARTPF1_Velvet--Singlet5661	9E-24	412	SUN6	1E-30	326	ethylene-responsive transcription factor ABI4 [Papaver somniferum]	2E-35	336
**EcAP2/ERF102**	182	MAGPIE:eca_ECARTPF1_Velvet--Contig6349	4E-18	201	Integrase-type DNA-binding superfamily protein	2E-35	187	AP2/ERF domain [Macleaya cordata]	3E-81	184
**EcAP2/ERF103**	196	MAGPIE:eca_ECARTPF1_Velvet--Contig6349	0	201	Integrase-type DNA-binding superfamily protein	1E-50	185	AP2/ERF domain [Macleaya cordata]	2E-74	188
**EcAP2/ERF104**	318	MAGPIE:eca_ECARTPF1_Velvet--Singlet37091	5E-89	326	Integrase-type DNA-binding superfamily protein	3E-34	298	AP2/ERF domain [Macleaya cordata]	2E-55	345
**EcAP2/ERF105**	301	MAGPIE:eca_ECARTPF1_Velvet--Contig10931	0	274	PLT2	1E-65	565	AP2-like ethylene-responsive transcription factor PLT2 isoform X2 [Papaver somniferum]	2E-117	279
**EcAP2/ERF106**	263	MAGPIE:eca_ECARTPF1_Velvet--Contig16505	0	266	PLT1	5E-71	571	AP2/ERF domain [Macleaya cordata]	7E-147	271
**EcAP2/ERF107**	406	MAGPIE:eca_ECARTPF1_Velvet--Contig3225	0	411	BBM	7E-69	580	AP2-like ethylene-responsive transcription factor PLT1 [Papaver somniferum]	1E-136	413
**EcAP2/ERF108**	548	MAGPIE:eca_ECARTPF1_Velvet--Singlet7487	0	376	AINTEGUMENTA-like 6	2E-85	543	AP2/ERF domain [Macleaya cordata]	2E-180	536
**EcAP2/ERF109**	617	MAGPIE:eca_ECARTPF1_Velvet--Singlet31985	0	568	CKC	8E-146	555	PREDICTED: AP2-like ethylene-responsive transcription factor AIL1 [Nelumbo nucifera]	1E-130	597

**EcAP2/ERF110**	503	MAGPIE:eca_ECARTPF1_Velvet--Contig23931	0	449	related to AP2.7	3E-97	464	PREDICTED: ethylene-responsive transcription factor RAP2-7-like isoform X1 [Nelumbo nucifera]	1E-142	511
**EcAP2/ERF111**	573	MAGPIE:eca_ECARTPF1_Velvet--Singlet31327	3E-50	699	PLT1	5E-67	571	PREDICTED: AP2-like ethylene-responsive transcription factor AIL1 [Nelumbo nucifera]	3E-149	597
**EcAP2/ERF112**	507	MAGPIE:eca_ECARTPF1_Velvet--Singlet7487	6E-129	376	AINTEGUMENTA-like 5	4E-128	558	AP2-like ethylene-responsive transcription factor AIL5 isoform X2 [Papaver somniferum]	2E-156	509
**EcAP2/ERF113**	552	MAGPIE:eca_ECARTPF1_Velvet--Singlet31327	6E-53	699	PLT2	2E-126	565	PREDICTED: AP2-like ethylene-responsive transcription factor PLT2 [Nelumbo nucifera]	4E-159	542
**EcAP2/ERF114**	373	MAGPIE:eca_ECARTPF1_Velvet--Singlet32373	0	171	Integrase-type DNA-binding superfamily protein	4E-10	307	PREDICTED: ethylene-responsive transcription factor ERF118 [Nelumbo nucifera]	1E-22	343
**EcAP2/ERF115**	593	MAGPIE:eca_ECARTPF1_Velvet--Singlet7856	1E-100	593	PLT2	8E-34	565	AP2/ERF domain [Macleaya cordata]	6E-80	543
**EcAP2/ERF116**	117	MAGPIE:eca_ECARTPF1_assembled--ECARTPF1_c8731	0.17	172	ethylene response factor 7	1E-06	244	AP2/ERF domain [Macleaya cordata]	7E-05	150
**EcAP2/ERF117**	674	MAGPIE:eca_ECARTPF1_Velvet--Singlet7856	1E-100	593	PLT2	6E-30	565	AP2-like ethylene-responsive transcription factor PLT2 isoform X2 [Papaver somniferum]	5E-82	555
**EcAP2/ERF118**	117	MAGPIE:eca_ECARTPF1_assembled--ECARTPF1_rep_c3805	0.59	139	ethylene response factor 7	2E-10	244	PREDICTED: ethylene-responsive transcription factor 9-like [Nelumbo nucifera]	5E-07	175
**EcAP2/ERF119**	369	MAGPIE:eca_ECARTPF1_Velvet--Singlet42715	0	368	AP2/B3 transcription factor family protein	1E-126	361	AP2/ERF and B3 domain-containing transcription factor RAV1-like [Papaver somniferum]	1E-169	379
**EcAP2/ERF120**	112	MAGPIE:eca_ECARTPF1_Velvet--Singlet38270	0.046	172	Integrase-type DNA-binding superfamily protein	1E-08	219	AP2/ERF domain [Macleaya cordata]	1E-06	150
**EcAP2/ERF121**	367	MAGPIE:eca_ECARTPF1_Velvet--Singlet41935	0	397	RAV1	1E-134	344	AP2/ERF and B3 domain-containing transcription factor RAV1-like [Papaver somniferum]	8E-157	387
**EcAP2/ERF122**	375	MAGPIE:eca_ECARTPF1_Velvet--Singlet7487	2E-56	376	Integrase-type DNA-binding superfamily protein	5E-84	430	AP2/ERF domain [Macleaya cordata]	9E-122	400
**EcAP2/ERF123**	314	MAGPIE:eca_ECARTPF1_Velvet--Singlet31985	3E-23	568	ARIA-interacting double AP2 domain protein	1E-23	211	AP2/ERF domain [Macleaya cordata]	9E-51	337
**EcAP2/ERF124**	564	MAGPIE:eca_ECARTPF1_Velvet--Contig5418	0	389	Integrase-type DNA-binding superfamily protein	3E-109	432	AP2-like ethylene-responsive transcription factor AIL5 isoform X2 [Papaver somniferum]	2E-155	604
**EcAP2/ERF125**	318	MAGPIE:eca_ECARTPF1_Velvet--Contig20512	3E-16	324	related to AP2 11	1E-29	253	AP2/ERF domain [Macleaya cordata]	7E-87	286

**EcAP2/ERF126**	373	MAGPIE:eca_ECARTPF1_Velvet--Singlet7678	0	166	cytokinin response factor 2	8E-19	343	AP2/ERF domain [Macleaya cordata]	3E-54	344
**EcAP2/ERF127**	227	MAGPIE:eca_ECARTPF1_Velvet--Contig12874	0	162	Integrase-type DNA-binding superfamily protein	3E-42	196	PREDICTED: ethylene-responsive transcription factor-like protein At4g13040 isoform X2 [Nelumbo nucifera]	3E-77	232
**EcAP2/ERF128**	132	MAGPIE:eca_ECARTPF1_Velvet--Contig25951	0.005	197	ERF domain protein 10	1E-09	245	PREDICTED: ethylene-responsive transcription factor 9-like [Nelumbo nucifera]	3E-07	175
**EcAP2/ERF129**	351	MAGPIE:eca_ECARTPF1_Velvet--Contig11750	0	351	Integrase-type DNA-binding superfamily protein	5E-38	341	PREDICTED: dehydration-responsive element-binding protein 2C-like [Nelumbo nucifera]	8E-56	362
**EcAP2/ERF130**	358	MAGPIE:eca_ECARTPF1_Velvet--Contig28265	0	355	hypothetical protein AXX17_AT2G37430	6E-34	194	AP2/ERF domain [Macleaya cordata]	5E-74	345
**EcAP2/ERF131**	306	MAGPIE:eca_ECARTPF1_Velvet--Contig20832	0.00000001	309	SUN6	1E-22	326	PREDICTED: ethylene-responsive transcription factor ABI4 [Nelumbo nucifera]	2E-20	290
**EcAP2/ERF132**	202	MAGPIE:eca_ECARTPF1_Velvet--Contig6349	3E-96	201	hypothetical protein AXX17_AT1G20160	1E-44	185	ethylene-responsive transcription factor ERF017-like [Papaver somniferum]	7E-60	209
**EcAP2/ERF133**	132	MAGPIE:eca_ECARTPF1_Velvet--Contig27787	0.0000007	203	Integrase-type DNA-binding superfamily protein	5E-13	219	AP2/ERF domain [Macleaya cordata]	6E-11	150
**EcAP2/ERF134**	132	MAGPIE:eca_ECARTPF1_Velvet--Singlet39940	0.0004	169	ethylene response factor 7	1E-10	244	PREDICTED: ethylene-responsive transcription factor 9-like [Nelumbo nucifera]	2E-08	175

Supplementary Table S5 Primer sequences

Gene		Oligonucleotide sequences (5' to 3')
EcAP2/ERF1	Fw	TGTGATTCCCCTTTTGATTTGG
	Rv	CACCACTGCCGCTTTAACTTTC
EcAP2/ERF2	Fw	CGGAATCTGACATCAAGCCTTC
	Rv	GCGATAGCAAGGGAAAGTCAAA
EcAP2/ERF3	Fw	AACACCTTCTCAGCGATTTTG
	Rv	TCTGTGACTTGGAAATTCTGTTGG
EcAP2/ERF4	Fw	TGAAAATGGCAACTCAAGACGA
	Rv	AGAAACCGCCGGAATTTCAA
EcAP2/ERF6	Fw	GCATTACTCAATTTCCCTGCTGA
	Rv	ATCATTTTTCGGCACGAGAGAG
EcAP2/ERF12	Fw	TACAGCCGAAGAGGCAGCTTTA
	Rv	CACCACAGGAGAACACCCTTCT
EcAP2/ERF16	Fw	TCGAGCACCGAATATCATCC
	Rv	CATTGAGACGAGGCAAGTCG
EcAP2/ERF17	Fw	GGATTCTTTTTCGGCCTTCC
	Rv	TGAGGCTTCTTTGGGTGCTT
EcAP2/ERF18	Fw	GCCAATTACACCACCAGCTCAT
	Rv	TGTTCTTGCTCGGGTTTCACTT
EcAP2/ERF19	Fw	GGACTGGCGATCTTCGATTC
	Rv	GACACCATTCCTTCTGCTAAAACA
EcAP2/ERF20	Fw	CATCACTACCCACGCAGCAC
	Rv	TATTTCGGCTGCCCATTTTC
EcAP2/ERF26	Fw	CAGCTCACCCACCACCAGTA
	Rv	CGATTCCACAAGTCCGACAA
EcAP2/ERF77	Fw	CCATGATGTAGCTGCACTTGC
	Rv	ACCGGCTTAAATAACATGGCC
EcAP2/ERF79	Fw	GCGGCGTTTTGTCTTAAAGG
	Rv	ACGTGGAAGGTCGAGGAAGA
EcAP2/ERF121	Fw	CCGGCCCTGATAAACAACTC
	Rv	TAACCACATGACCCGTTGGA
EcbHLH1-1	Fw	CTAGACAGATCGCCTGTTTTGTTG
	Rv	GAGATAGATGACCCTTTAGATAATGAGATCC
EcbHLH1-2	Fw	GGTTGGACAAATCACCAGCTTTAC
	Rv	AAGATAGAGGGGTTCATAATGATAGCAAG

Ec6OMT	Fw	CCTGTTCAACCCGTTGACTTAG
	Rv	CCCAACCCTTAATCAGAAATTTG
EcCYP719A5	Fw	CCTGATCTTAGTGAGGATCATTGC
	Rv	ATGCTAGCACTACATGCCATTTCAC
EcActin	Fw	GGTATTGTGCTGGATTCTGGTG
	Rv	GTAGGATTGCGTGGGGTAGTG

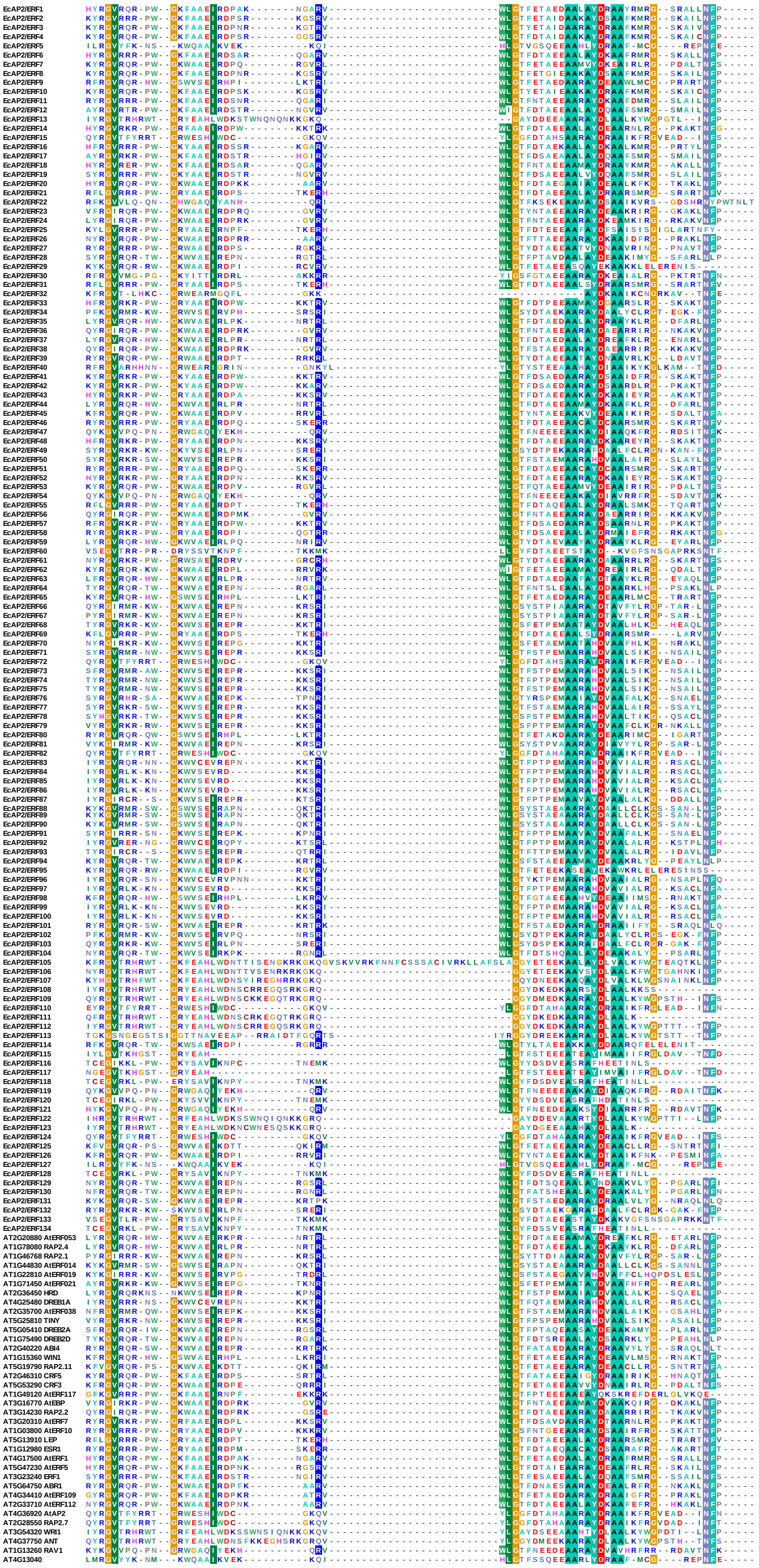

Supplementary Figure S1

Multiple sequence alignment of AP2/ERF domain of *E. californica* and *A. thaliana* AP2/ERF transcription factors.

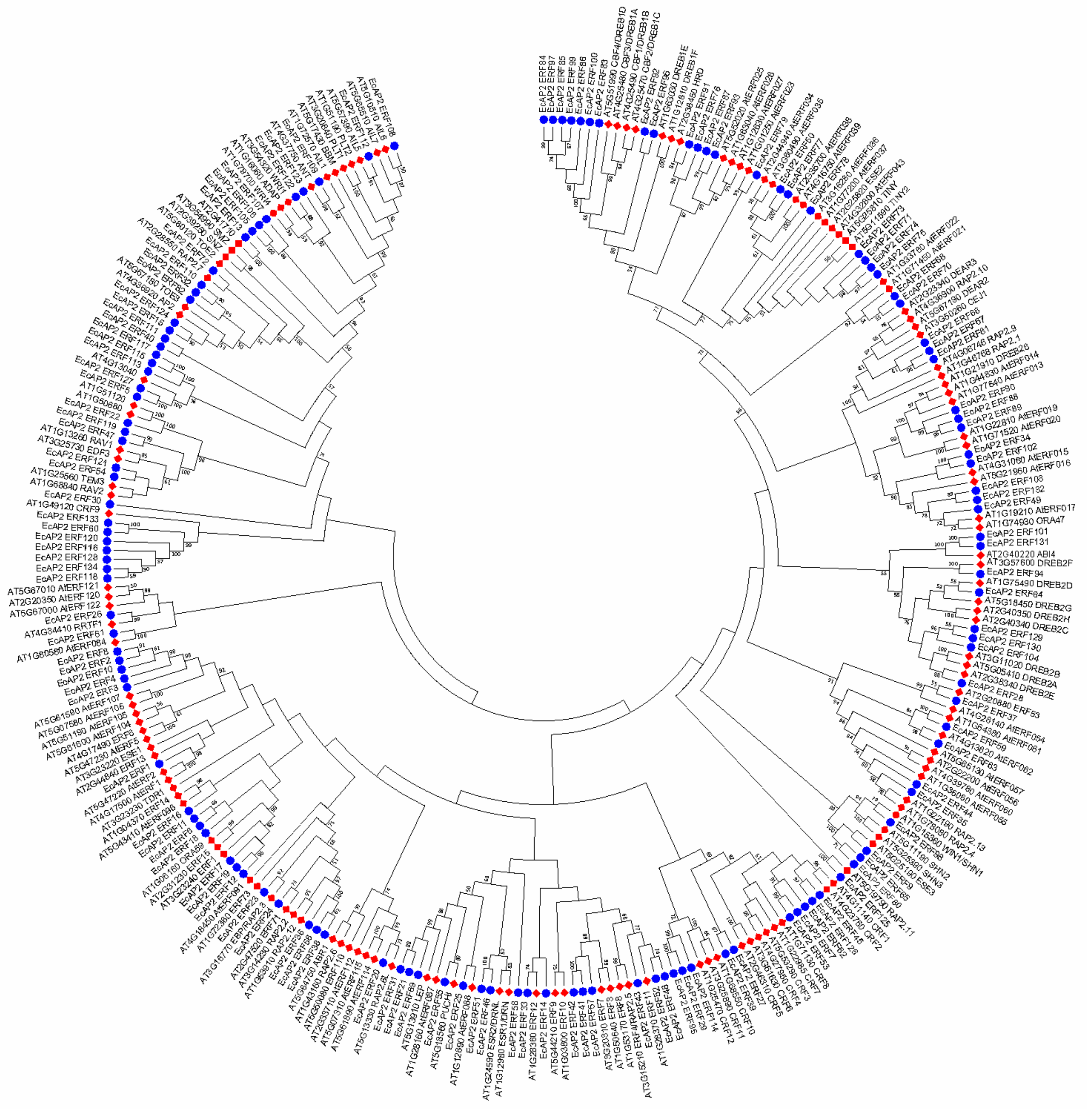

Supplementary Figure S2

Un-rooted phylogenetic tree of full-length *E. californica* and *A. thaliana* AP2/ERF transcription factors.

The NJ tree was constructed from the full-length amino acid sequences of the 134 *Ec*AP2/ERF (blue circle) and 147 Arabidopsis AP2/ERF (red diamond) proteins. Bootstrap confidence values from 1000 replicates are indicated at each branch.
